# Review: The evolution of peptidergic signaling in Cnidaria and Placozoa, including a comparison with Bilateria

**DOI:** 10.3389/fendo.2022.973862

**Published:** 2022-09-23

**Authors:** Frank Hauser, Thomas L. Koch, Cornelis J. P. Grimmelikhuijzen

**Affiliations:** ^1^ Section for Cell and Neurobiology, Department of Biology, University of Copenhagen, Copenhagen, Denmark; ^2^ Department of Biomedical Sciences, University of Copenhagen, Copenhagen, Denmark

**Keywords:** peptide, receptor, evolution, G protein-coupled receptor (GPCR), leucine-rich repeat-containing GPCR (LGR), DEG/ENaC, nervous system, endocrine system

## Abstract

Bilateria have bilateral symmetry and are subdivided into Deuterostomia (animals like vertebrates) and Protostomia (animals like insects and mollusks). Neuropeptides occur in both Proto- and Deuterostomia and they are frequently structurally related across these two lineages. For example, peptides belonging to the oxytocin/vasopressin family exist in both clades. The same is true for the G protein-coupled receptors (GPCRs) of these peptides. These observations suggest that these neuropeptides and their GPCRs were already present in the common ancestor of Proto- and Deuterostomia, which lived about 700 million years ago (MYA). Furthermore, neuropeptides and their GPCRs occur in two early-branching phyla that diverged before the emergence of Bilateria: Cnidaria (animals like corals and sea anemones), and Placozoa (small disk-like animals, feeding on algae). The sequences of these neuropeptides and their GPCRs, however, are not closely related to those from Bilateria. In addition, cnidarian neuropeptides and their receptors are not closely related to those from Placozoa. We propose that the divergence times between Cnidaria, Placozoa, and Bilateria might be too long for recognizing sequence identities. Leucine-rich repeats-containing GPCRs (LGRs) are a special class of GPCRs that are characterized by a long N-terminus containing 10-20 leucine-rich domains, which are used for ligand binding. Among the ligands for LGRs are dimeric glycoprotein hormones, and insulin-like peptides, such as relaxin. LGRs have been found not only in Proto- and Deuterostomia, but also in early emerging phyla, such as Cnidaria and Placozoa. Humans have eight LGRs. In our current review, we have revisited the annotations of LGRs from the sea anemone *Nematostella vectensis* and the placozoan *Trichoplax adhaerens*. We identified 13 sea anemone LGRs and no less than 46 LGRs from *T*. *adhaerens*. All eight human LGRs appear to have orthologues in sea anemones and placozoans. LGRs and their ligands, therefore, have a long evolutionary history, going back to the common ancestor of Cnidaria and Placozoa.

## 1 Introduction: Neuropeptides and their GPCRs in Bilateria

**Figure 1:**
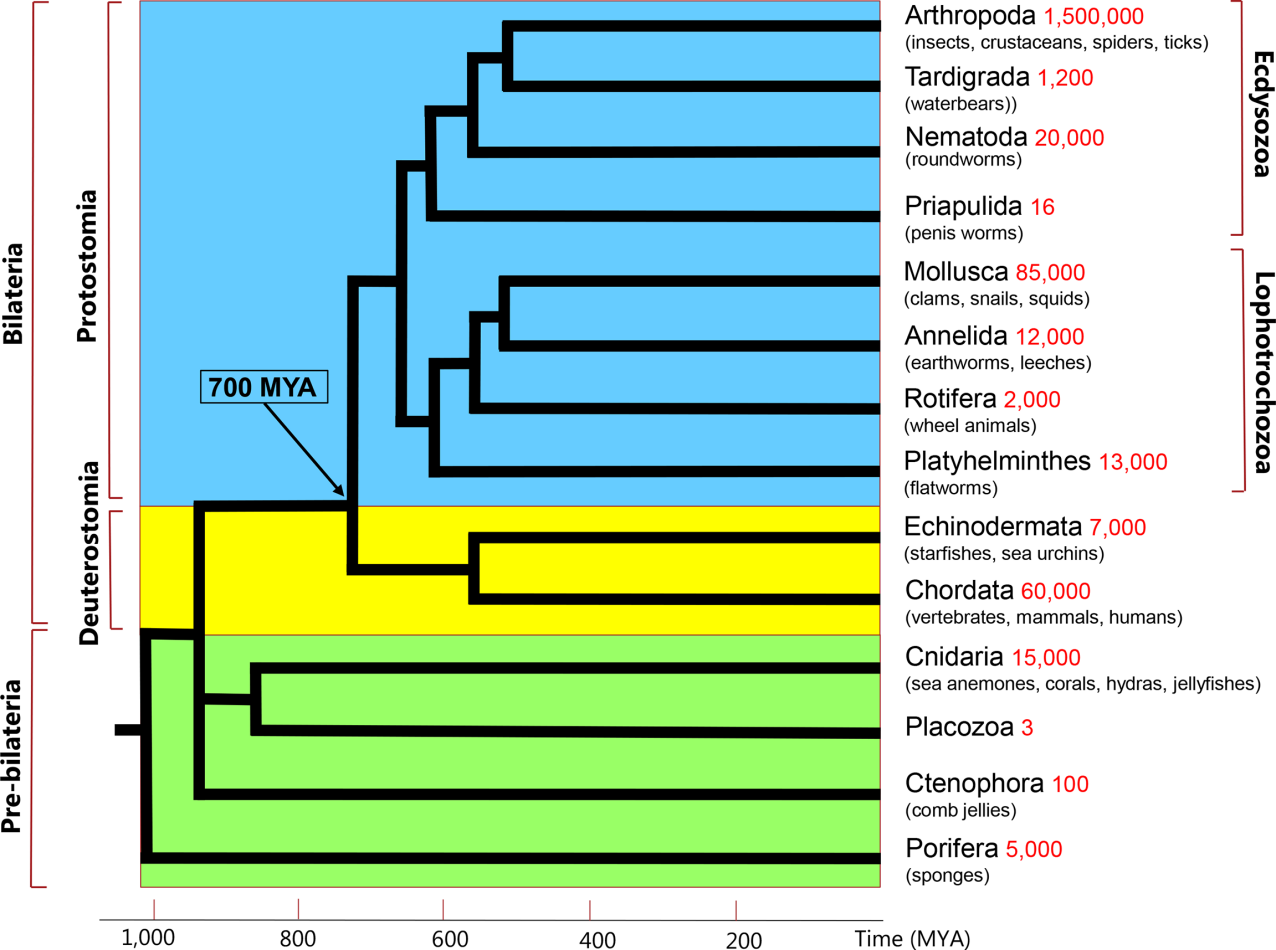
Simplified phylogenetic tree of metazoans. These animals can be subdivided into Protostomia (branches with blue background), and Deuterostomia (braches with yellow background). The Proto- and Deuterostomia originated 700 million years ago (MYA), see arrow and time scale on the X-axis. These two taxa together are also called Bilateria, because of their bilateral symmetry. There are four phyla (Cnidaria, Placozoa, Ctenophora, Porifera) that diverged before the emergence of Bilateria (branches with green background). Two of these early-branching phyla, the Cnidaria and Placozoa, which probably form a monophyletic clade, are discussed in the current review. The phylogenetic position of Ctenophora is still uncertain. The phyla listed in the right column are extant phyla and do not represent all extant animal phyla. Their estimated species numbers are given in red.

Neuropeptides have a broad distribution in the animal kingdom and a long evolutionary history, occurring in both Deuterostomia (animals like mammals and other chordates) and Protostomia (animals like insects, crustaceans, mollusks, round- and flatworms) (**Figure 1**) (1, 2). The Proto- and Deuterostomia diverged about 700 million years ago (MYA) (3), which also marked the birth of the Bilateria (animals with a bilateral symmetry) (**Figure 1**). When comparing the neuropeptides from Proto- and Deuterostomia, we can occasionally observe that their sequences are conserved (1, 2, 4, 5). Examples of such conserved sequences between the two evolutionary lineages are peptides belonging to the oxytocin/vasopressin family, neuropeptide-Y family, gastrin/cholecystokinin (CCK)-like peptides, and tachykinin-like peptides (1, 2, 4–10). The existence of identical or nearly identical neuropeptide families in Proto- and Deuterostomia suggests that these peptides originated in the common ancestor of these two evolutionary lineages.

For some of the neuropeptide families, their members have striking sequence identities in Proto- and Deuterostomia, such as members of the oxytocin/vasopressin neuropeptide family, where six out of nine amino acid residues are identical, including an essential cystine bridge between Cys-1 and Cys-6 of the peptide sequence (7). Many chordate gastrin/CCKs have YGWMDFamide, YMGWMDFamide, or YYGWMDFamide as their C-terminal sequences, where the Y residues are sulfated (10). Sulfation is unusual in neuropeptides and is a hallmark for chordate gastrin/CCKs (10). Protostomes also have gastrin/CCK-like peptides called sulfakinins. For example, the cockroach *Leucophea maderae*, which was the first insect from which sulfakinins have been isolated, has a sulfakinin peptide with the C-terminal sequence YGHMRFamide. This peptide has a sulfated Y residue at position 6 counted from the C-terminus, which corresponds to the positions of the sulfated Y residues in chordate gastrin/CCKs (8, 9). The cockroach C-terminal six amino acid residue sequence has four amino acid residues in common with mammalian gastrin/CCK, including the characteristic sulfated Y residue and is, thus, a likely orthologue of the mammalian peptide. For many other protostome neuropeptides, however, the situation is not clear-cut and other independent methods are required to establish robust evolutionary relationships between neuropeptide families in Proto- and Deuterostomia. One powerful method in this context is to look at the neuropeptide receptors, because, in our experience, neuropeptide receptors are much more conserved than their neuropeptide ligands. Furthermore, receptors are much larger (about 350 amino acid residues) than their smaller neuropeptide ligands (3-20 residues) and are, thus, easier to identify, using bioinformatics tools (11, 12).

**Figure 2:**
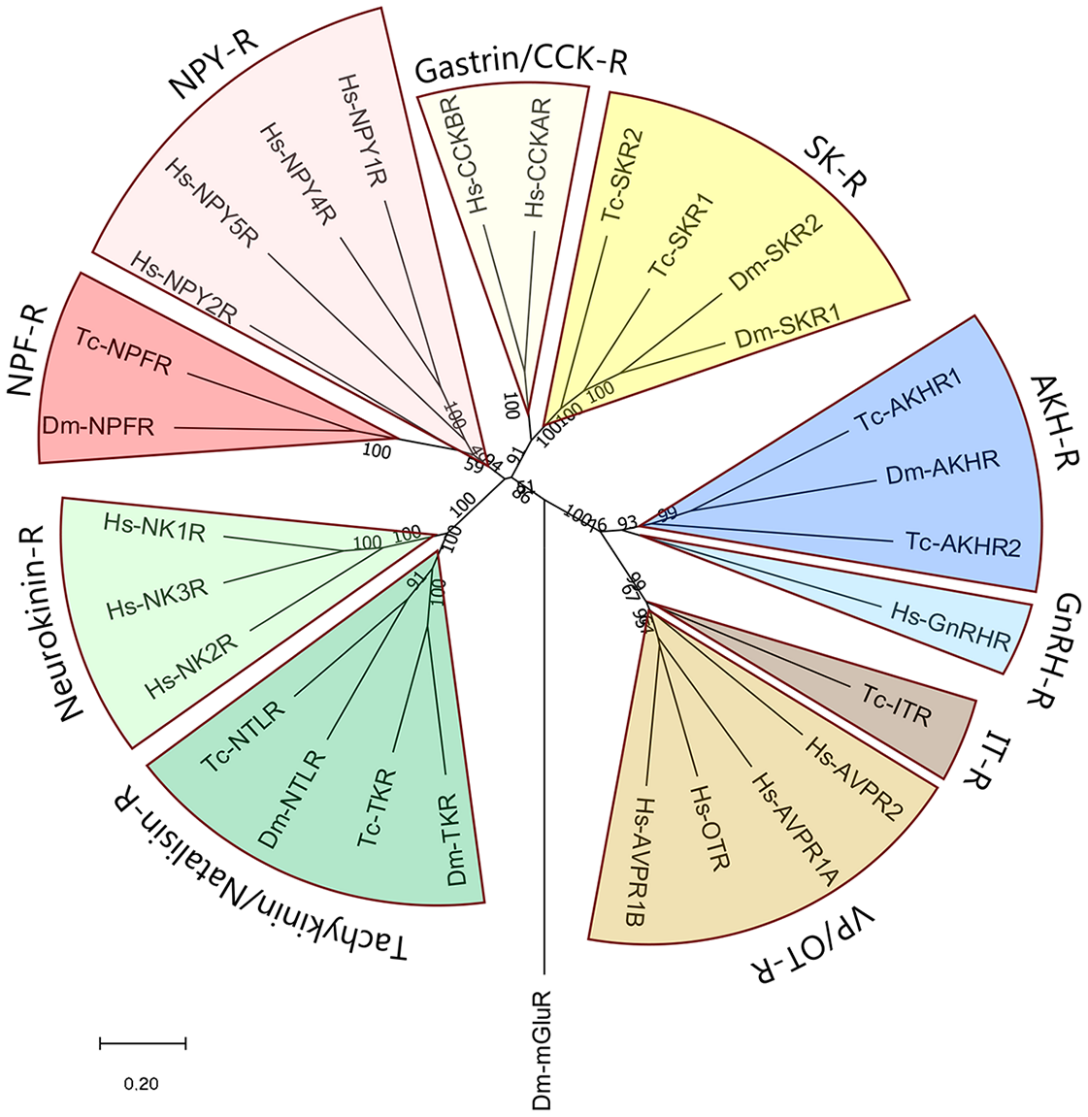
Phylogenetic tree of some neuropeptide GPCRs that have orthologues in both proto- and deuterostomes. As representatives of the deuterostomes we have taken humans (*Homo sapiens*, abbreviated *Hs*). As representatives of the protostomes, we have selected two model insects: The fruit fly *Drosophila melanogaster* (abbreviated *Dm*), and the red flour beetle *Tribolium castaneum* (abbreviated *Tc*). The figure shows that the two sulfakinin receptors (SK-R) found in each of these two insects are the orthologues of the two human gastrin/CCK receptors (gastrin/CCK-R); that the insect AKH receptors (AKH-R) are the orthologues of the human GnRH receptors (GnRH-R); that the insect oxytocin/vasopressin (= inotocin) receptors (IT-R) are the orthologues of the human vasopressin/oxytocin receptors (VP/OT-R); that the insect tachykinin/natalisin receptors are the orthologues of the human neurokinin receptors; and that the insect neuropeptide-F receptors (NPF-R) are the orthologues of the human neuropeptide-Y receptors (NPY-R). The numbers associated with the branches are bootstrap support values. The tree is rooted with the metabotropic glutamate receptor CG11144 from *D. melanogaster* (Dm-mGluR). The accession numbers of the GPCR sequences used to calculate this tree are provided in **Supplementary Table S1**.

Most neuropeptide receptors are G protein-coupled receptors (GPCRs). Many of these receptors have nowadays been deorphanized (= “matched” with their neuropeptide ligands), both in Protostomia (13, 14) and Deuterostomia (15, 16). Thus, when neuropeptide orthologues are claimed between the two bilaterian evolutionary lineages (**Figure 1**), one should always try to test these claims by showing the presence of orthologous GPCRs. Indeed, the identifications of oxytocin/vasopressin, neuropeptide Y, gastrin/CCK, and tachykinin peptides in Protostomia, have always been followed up by the subsequent identifications of their cognate GPCRs in this animal taxon and the demonstrations that these GPCRs are the orthologues of their deuterostome counterparts (4–7, 17–24). **Figure 2** shows that the protostome (in this case, insect) oxytocin/vasopressin, neuropeptide Y, gastrin/CCK, and tachykinin GPCRs are the orthologues of their deuterostome (in this case, human) counterparts. These findings confirm that the complete ligand/receptor couples have been conserved during 700 million years of evolution in a process of ligand/receptor co-evolution.

In the following, we would like to give an example, where we could recognize two gonadotropin-releasing hormone (GnRH) receptor orthologs, one in Deuterostomia and the other one in Protostomia, but where we initially were unable to identify the ligand for the protostome GPCR. GnRH is a neuropeptide produced by a population of neuroendocrine cells in the vertebrate hypothalamus, where it stimulates the release of the glycoprotein hormones, luteinizing hormone (LH), and follicle stimulating hormone (FSH) from the anterior pituitary (2). GnRH and the two glycoprotein hormones are essential for vertebrate reproduction (2). To gain more insight into the reproduction of the fruitfly *Drosophila melanogaster*, we cloned a GPCR from this fruitfly that was a clear orthologue of the mammalian GnRH receptor (25). Next, we started to search for the *D. melanogaster* GnRH receptor ligand, which we expected would be GnRH or a GnRH-like peptide. However, we were unsuccessful with finding such a peptide, even after the publication of the *Drosophila* genome sequence, two years later (26), and carrying out bioinformatic searches. Therefore, we started a project where we purified the *Drosophila* GnRH receptor ligand from an aqueous extract of *Drosophila* larvae, using second messenger production in Chinese Hamster Ovary (CHO) cells that were expressing the *Drosophila* GnRH receptor, as a bioassay (27). This project resulted in the isolation and sequencing of a neuropeptide, which, to our surprise, was adipokinetic hormone (AKH), an insect neuropeptide, which had been known for more than twenty-five years (28), but which had an amino acid sequence that was completely different from mammalian GnRH (27). AKH is a metabolic neuropeptide that mobilizes lipids and carbohydrates from the insect fat body during a flight and other energy-requiring activities (28). Thus, not only are the structures of the mammalian GnRH and insect AKH completely different, but also are their actions. Yet, the presence of the two orthologous receptors in Proto- and Deuterostomia implies that these two receptors must have had a common ancestor that evolved before the split of Proto- and Deuterostomia at 700 MYA (**Figure 1**).

In conclusion, the following main lessons can be learned from the examples described in this chapter 1: (i) Many bilaterian neuropeptides and their GPCRs evolved in the common ancestor of Proto- and Deuterostomia, which lived around 700 MYA; (ii) Neuropeptide GPCRs are often more conserved than their ligands. Identifying (= deorphanizing) neuropeptide GPCRs may reveal evolutionary trajectories that otherwise would stay obscured (example: GnRH and AKH receptors).

## 2 Biosynthesis of neuropeptides

Neuropeptides are synthesized as proteins (preprohormones) on the ribosomes of the rough endoplasmic reticulum (RER). After translation and during transport across the RER membrane, the pre-part of the preprohormone (also called signal peptide) is removed by a signal peptidase, after which the prohormone is directed into the secretory pathway. Here, the prohormones are packed into dense-core vesicles together with at least four prohormone processing enzymes, which catalyse the conversion of the prohormones into biologically active neuropeptides: (i) Prohormone convertase 1/3 (PC 1/3) that cleaves C-terminally of R, RR, or KR sequences. The resulting peptide products are still C-terminally elongated by R, RR, or KR residues. (ii) These C-terminal residues are removed by a carboxypeptidase specific for basic residues. (iii) About 50% of all neuropeptides carry a C-terminal amide group, which protects against C-terminal enzymatic degradation. This C-terminal amide group is formed from a C-terminal Gly residue with the help of peptidylglycine alpha-monooxygenase. (iv) Many neuropeptides carry an N-terminal pGlu residue, which protects the peptide against N-terminal enzymatic degradation. This N-terminal pGlu residue is formed by cyclization of an N-terminal Gln residue catalyzed by the enzyme glutaminyl cyclase. In a few cases, neuropeptides have N-terminal XP, or XPP sequences, which have a secondary amine, instead of a primary amine, involved in the peptide bond, which also protects against N-terminal enzymatic degradation (29–32).

## 3 Neuropeptides and their GPCRs in Cnidaria

Neuroendocrine systems evolved in a group of ancient animals that originated before the split of Proto- and Deuterostomia (**Figure 1**). These “pre-bilaterian” animals were related to four extant animal phyla: Porifera (sponges); Ctenophora (comb jellyfishes); Placozoa (1-mm small, disk-like animals, feeding on algae); and Cnidaria (animals like sea anemones, corals, and jellyfishes). Only Cnidaria and Placozoa will be discussed in this review, because they probably form a monophyletic clade (explained below) and cnidarians have well-established peptidergic nervous systems. A recent comment on neuropeptides and nervous systems in Ctenophora can be found in (33).

**Figure 3:**
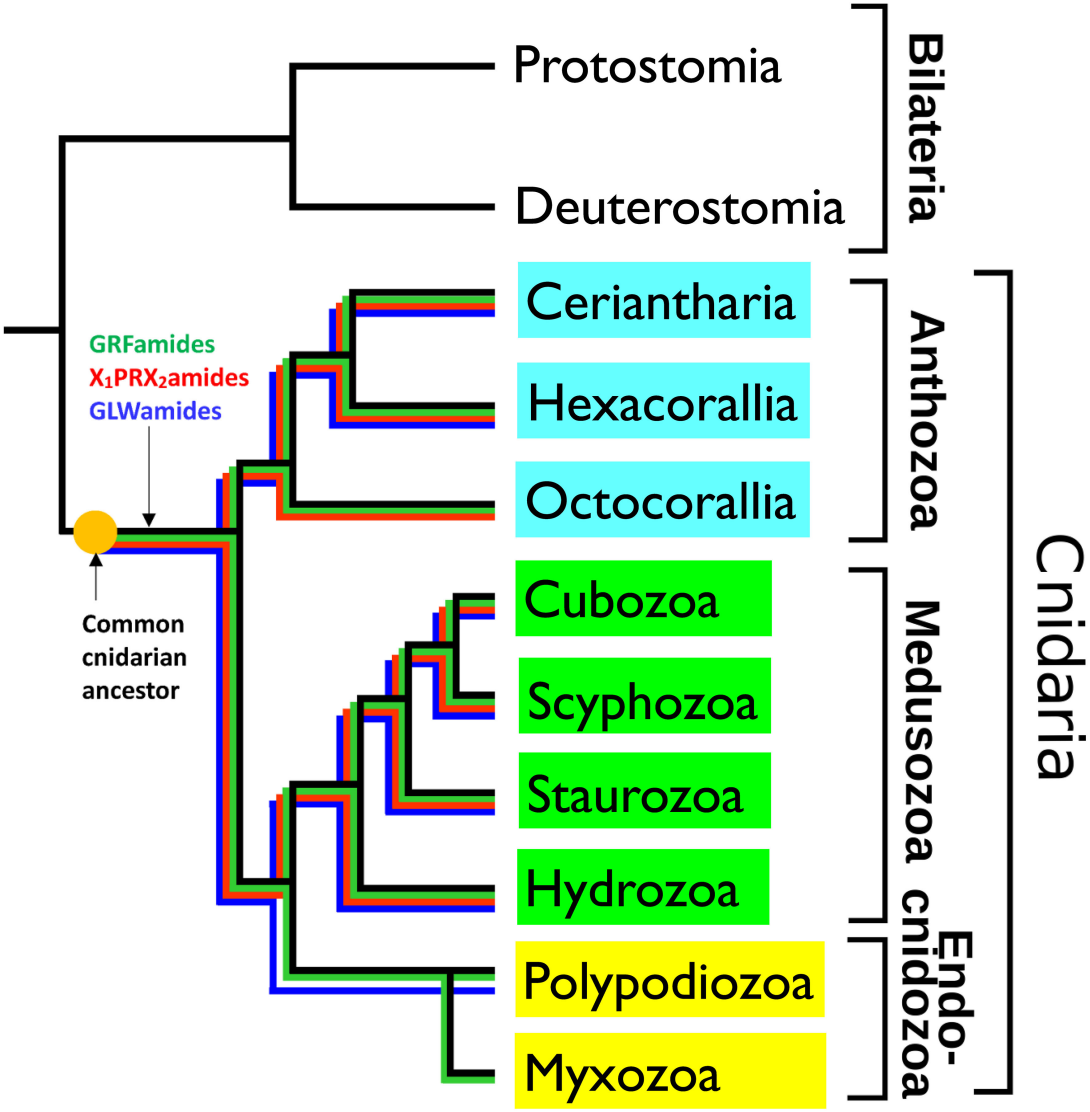
Schematic drawing, showing the phylogenetic positions of the cnidarian subclasses Ceriantharia, Hexacorallia and Octocorallia (belonging to the subphylum Anthozoa, highlighted in blue), the classes Cubozoa, Scyphozoa, Staurozoa, Hydrozoa (belonging to the subphylum Medusozoa, highlighted in green), and Polypodiozoa, and Myxozoa (belonging to the subphylum Endocnidozoa, highlighted in light yellow). The figure also shows the presence of three ancestral neuropeptide families in these clades by colored lines: the X_1_PRX_2_amide family (highlighted in red), the GRFamide family (highlighted in green), and the GLWamide family (highlighted in dark blue). These neuropeptide families are present in all the investigated Ceriantharia, Hexacorallia, Cubozoa, Scyphozoa, Staurozoa, and Hydrozoa species. However, the Octocorallia have apparently lost their GLWamide genes. The endoparasitic Polypodiozoa and Myxozoa are strongly reduced. Only a few species produce GRFamide and GLWamide peptides, while in all endocnidozoan species the X_1_PRX_2_amide peptides are absent. The presence of the three neuropeptide families in all the other cnidarian classes and subclasses suggests that they also were present in the common cnidarian ancestor. Adapted from (34) with permission.

Cnidarians are a diverse group of animals characterized by having cnidocytes, which are stinging cells used for capturing prey. The phylum Cnidaria is sub-divided into several classes and subphyla (**Figure 3**). These are the subphylum Anthozoa, which includes sea anemones and corals; the subphylum Medusozoa, which consists of the classes Cubozoa (box jellyfishes), Scyphozoa (true jellyfishes), Staurozoa (stalked jellyfishes), and Hydrozoa (animals like *Hydra* and hydromedusae); and the subphylum/class Endocnidozoa, which are small endoparasites, belonging to two taxa, the Polypodiozoa, and Myxozoa. In the past, neuropeptides have only been isolated and sequenced from just a few model cnidarians, like *Hydra magnipapillata*, *Clythia hemisphaerica*, and the sea anemone *Anthopleura elegantissima* (35–55). These peptides are 4-10 amino acid residues long and are protected at their C-termini by amide groups against carboxypeptidases, and at their N-termini by either pGlu (pQ), XP, or XPP residues against aminopeptidases. Thus, the overall organization of these neuropeptides resembles the overall structures of the neuropeptides isolated from Bilateria. Yet, with respect to the actual amino acid sequences, these isolated cnidarian neuropeptides had no homologues in the Bilateria. We and other researchers raised antibodies against the various isolated cnidarian neuropeptides and used them for whole-mount immunocytochemistry. This procedure enabled us to stain the cnidarian nervous systems with unprecedented details (35–37, 56–61).

Cnidarian neuropeptides control physiological processes like reproduction, development, metamorphosis, feeding, and muscle contraction (35–37, 46, 49–51, 53–55, 62–64).

We and other research groups also cloned the cnidarian neuropeptide preprohormones (35–37, 65–71). These preprohormones often have very high copy numbers of the immature neuropeptides. For example, the *A. elegantissima* preprohormone for Antho-RFamide has 21 copies of Antho-RFamide (pQGRFamide) and the Antho-RFamide preprohormone from the sea anemone *Calliactis parasitica* has 19 copies of this neuropeptide sequence (65, 66). At the C-terminal sides of each of the immature cnidarian neuropeptide sequences, we found classical processing signals for PC1/3 and, as in Bilateria, C-terminal Gly residues, which are converted into amide groups (35–37, 65–71). We also cloned the sea anemone peptidylglycine alpha-monooxygenase and determined its intron/exon organization, showing that this enzyme is evolutionarily closely related to its bilaterian counterparts (31, 72, 73). Thus, cnidarian preprohormone processing is “classical” at the C-terminal sites of their immature neuropeptide sequences. To our surprise, however, we found that processing at the N-terminal sites of the neuropeptide sequences was rather different, because no basic residues were present for cleavage by PC1/3. Instead, acidic residues (D, E) and sometimes other residues were flanking the N-termini of the immature neuropeptides. In the preprohormone for the isolated and sequenced sea anemone neuropeptide Antho-RFamide (pQGRFamide), for example, we find 14 copies of the immature sequence DQGRFGKR and 5 copies of EQGRFGKR (65). We do not know the identity of the N-terminal processing enzyme, which could either be an aminopeptidase or endoproteinase. Unorthodox N-terminal processing of neuropeptide sequences appears to be specific for ancient peptidergic nervous and endocrine systems. This type of processing is not present in Bilateria, but, interestingly, it also seems to occur in Placozoa (see Chapter 4). These findings would suggest a close phylogenetic relationship between Cnidaria and Placozoa.

Cnidaria consists of more than 15,000 species that are distributed over the nine classes or subclasses shown in **Figure 3**. As mentioned above, neuropeptides from only five cnidarian species have been isolated and sequenced (35–55). Therefore, we wanted to know, whether the other cnidarians also produced neuropeptides and what the structures of these neuropeptides were. We assumed that investigating this question might lead to the discovery of novel neuropeptides with novel actions, thereby promoting cnidarian biology. In addition, this work might shed light on the evolution of neuropeptides in Cnidaria and tell us more about the neuropeptides that existed in the common cnidarian ancestor. To answer these questions, we investigated the sequenced genomes and transcriptomes from 80 cnidarian species, belonging to different families of the Ceriantharia, Hexacorallia, Octocorallia, Cubozoa, Scyphozoa, Staurozoa, Hydrozoa, Polypodiozoa, and Myxozoa (**Figure 3**) (34, 74–77).

For screening this large number of genomes and transcriptomes, we developed a bioinformatics script that recognized proteins, containing at least three repetitive sequences, each having the C-terminal sequence GKR, GRR, or GR (34, 74–77). In addition, we applied TBLASTN screening with queries corresponding to known cnidarian, placozoan, and bilaterian neuropeptide sequences (34, 74–77). We found that most cnidarians produced three to eight neuropeptide families. Nearly all cnidarian species produced peptides with the C-terminal sequence GRFamide, GLWamide, or X_1_PRX_2_amide (**Figure 3**). The only two exceptions were species belonging to the Octocorallia, where apparently a gene loss has occurred of their GLWamide peptide genes, and Endocnidozoa, where only a few species produced GRFamide, and GLWamide peptides, while the X_1_PRX_2_amides were lacking (**Figure 3**). Due to their endoparasitic lifestyle, endocnidozoans often consist of just a handful of cells and they have one of the smallest genomes in the animal kingdom. These strongly reduced genomes probably explain the gene losses of some of their neuropeptide genes (34).

The presence of GRFamides, GLWamides, and X_1_PRX_2_amides in nearly all cnidarian species clearly shows that these three neuropeptide families must have been present in the common cnidarian ancestor (**Figure 3**). They belong, thus, to the most ancient neuropeptide families known, so far.

The amino acid sequences of the three primordial neuropeptide families can vary somewhat, although their hallmarks are conserved. For the GRFamide family, for example, hexacoral and octocoral species (**Figure 3**) all have a short peptide version: pQGRFamide (75, 76). In hydrozoans, the GRFamides are N-terminally elongated. For instance, C. *hemisphaerica* contains a preprohormone, with 17 copies of the neuropeptide pQWLNGRFamide (34).

Some cnidarians have only one gene coding for GRFamides, one gene for GLWamides, and one gene for X_1_PR_2_Xamide peptides, such as the hydrozoans *Dynamena pumila*, *Porpita porpita*, *Vellela vellela*, and *Millipora alconis* (34). These three neuropeptide genes are apparently sufficient for a hydrozoan animal to live its daily hydrozoan life, including reproduction, development, metamorphosis, and feeding. Other cnidarians have duplicated one or more of these primordial genes, assumedly to give them a more advanced neuropeptide repertoire, which enables them to carry out a more advanced behavior. An extreme example of such gene multiplications can be observed in the freshwater hydromedusa *Craspedacusta sowerbii*, which has six GRFamide preprohormone genes, three GLWamide preprohormone genes, and two X_1_PRX_2_amide preprohormone genes (34). Furthermore, *C. sowerbii* has created an additional novel gene for a preprohomone that contains two copies of pQFLRPamide and one copy of pQFIRPamide (34). The presence of twelve neuropeptide preprohormone genes in *C. sowerbii* (compared to only three) is surprising and we do not know the reason for this.

Normally, cnidarians have genes for each of the three primordial neuropeptide families and, in addition, zero to three additional neuropeptide genes. These additional neuropeptide genes are taxon-specific and we assume that the animals need them for group-specific behaviour or physiology.

Do the current cnidarian neuropeptide genes have orthologues in the Bilateria and could we perhaps follow their evolution from pre-bilaterians to bilaterians? We have investigated this question by TBLASTN screening of the genomes and transcriptomes from species belonging to the two animal groups and found little or no resemblance between cnidarian and bilaterian neuropeptides or preprohormones. The best candidates for having orthologues in cnidarians and bilaterians would be members of the RFamide peptide family. In the following, therefore, we would like to focus on the RFamide neuropeptide family. As mentioned earlier in this chapter, RFamide peptides are abundant in nearly all cnidarians, where they have the C-terminal sequence GRFamide, which is N-terminally elongated by one to four amino acid residues (34, 75, 76). RFamide peptides are also occurring in many protostomes and to a lesser degree in deuterostomes. In **Supplementary Figure S1**, we have aligned the amino acid sequences of RFamide peptides from some cnidarian species (*N. vectensis, H. magnipapillata*) with those from several protostomes (the mollusc *Lymnaea stagnalis*; the brachiopod *Terebratalia transversa*; the nematode *Caenorhabditis elegans*; the fruitfly *Drosophila melanogaster*) and deuterostomes (the lancelet *Branchiostoma floridae*; and *Homo sapiens*). The results from these alignments were that, of course, all neuropeptides had the RFamide sequence in common, but that the N-terminal extensions were different. The structural similarities between the peptides were, therefore, very few and limited to the last two amino acid residues. We also compared the RFamide peptides, using phylogenetic tree analyses, which yielded trees with low bootstrap values and unreliable phylogenetic relationships, except for the cnidarian peptides (**Supplementary Figure S1**). Next, we compared the preprohormones of all the selected RFamides. This showed that some of the protostome RFamide preprohormones had a similar overall structure as the cnidarian RFamide preprohormones with a high number of immature RFamide neuropeptide sequences arranged in a repetitive manner directly after each other. In deuterostomes, these highly repetitive RFamide sequences were generally absent, such as in the kisspeptin-preprohormone and structurally related vertebrate preprohormones (**Supplementary Figure S1**) (78). We also analysed these preprohormones using phylogenetic tree analyses, which resulted, again, in trees with low bootstrap values and unreliable phylogenetic relationships. (**Supplementary Figure S1**). Based on these results, therefore, we cannot conclude that bilaterian RFamide neuropeptides are phylogenetically related to the cnidarian RFamide neuropeptides.

In analogy to the GnRH/AKH receptor story in Bilateria (Chapter 1), the amino acid sequences of GPCRs may be more conserved than the amino acid sequences of their neuropeptide ligands in Cnidaria. Finding GPCR orthologues in Cnidaria and Bilateria, followed by deorphanization, could help to identify orthologous GPCR/neuropeptide couples and give us useful information about their evolution (78). GPCR conservation between cnidarians and bilaterians has been investigated by Thiel et al. (79), but they were unable to find GPCR orthologues between the two animal groups. In our current paper, we extended this approach by comparing virtually all neuropeptide GPCRs from humans, the model sea anemone *N. vectensis* and the placozoan *T. adhaerens* (see Chapter 4) in a large phylogenetic tree analysis. GPCRs can be subdivided in three major classes: Family-A, -B, and -C, where family-A (also called rhodopsin-like GPCRs) is the largest class, comprising, among others, neuropeptide receptors, glycoprotein hormone receptors, olfactory receptors, and the opsins. We, therefore, collected the sequences from 723 human family-A receptors and removed the 437 olfactory receptors known to exist in humans, resulting in 286 human family-A receptors and compared them with 843 family-A GPCRs from *N. vectensis* and 679 family-A GPCRs from *T*. *adhaerens* (altogether 1826 receptors) in phylogenetic tree analyses. These analyses are described in **Supplementary Figures S3** and **S4**. They showed that we were unable to identify the existence of neuropeptide GPCR orthologues in the three species in a convincing way, except for a group of special GPCRs, the leucine-rich repeats-containing GPCRs, which we will discuss in Chapter 5.

So far, only one neuropeptide GPCR has been deorphanized in cnidarians, which is the receptor for an oocyte maturation inducing hormone (MIH) from the hydromedusa *Clytia hemisphaerica* (80). We found, however, that also this MIH GPCR had no convincing orthologues in bilaterians, in *N. vectensis*, and in *T. adhaerens* (as tested by TBLASTN).

Finding neuropeptide GPCR orthologues between bilaterians, cnidarians, and placozoans might be hard, due to the long divergence times, existing between these three animal clades. In addition, deorphanizing the large number of family-A GPCRs in cnidarians or placozoans would also be a huge task, as it would require the molecular cloning and expression of around 800 cnidarian or placozoan GPCRs, followed by functional expression of their cDNAs in human cell lines and screening of neuropeptide libraries. However, a recent publication shows that there are possibilities for identifying neuropeptide-GPCR couples by machine learning-assisted strategies, which potentially would shortcut the first labor-intensive steps (81, 82).

Recently, the precursor of a presumed neuropeptide, phoenixin, was reported to occur in bilatarians, cnidarians, ctenophores, and sponges (83). However, a cognate GPCR for this peptide could not be identified in any of the four animal clades (83).

## 4 Peptides and their GPCRs in Placozoa

In 1883, F.E. Schulze, a German zoologist at the University of Graz (Austria), discovered tiny animals, feeding on algae in a sea water aquarium and named them *Trichoplax adhaerens* (84, 85). *T. adhaerens* has remained the only representative of the phylum Placozoa during more than 130 years, but in the last few years, several other placozoan species or strains were isolated from marine waters from different parts of the globe (85). So far, the genomes from three species/strains have been sequenced: *T. adhaerens* (strain H1), *T. adhaerens* (strain H2), and *Hoilungia hongkongensis* (85). Nikitin identified twelve peptide preprohormones in *T. adhaerens* (strain H1) (86). We repeated his experiments in all three placozoan species/strains, using the script described in Chapter 3 and could confirm the existence of all twelve preprohormones (77).

**Figure 4:**
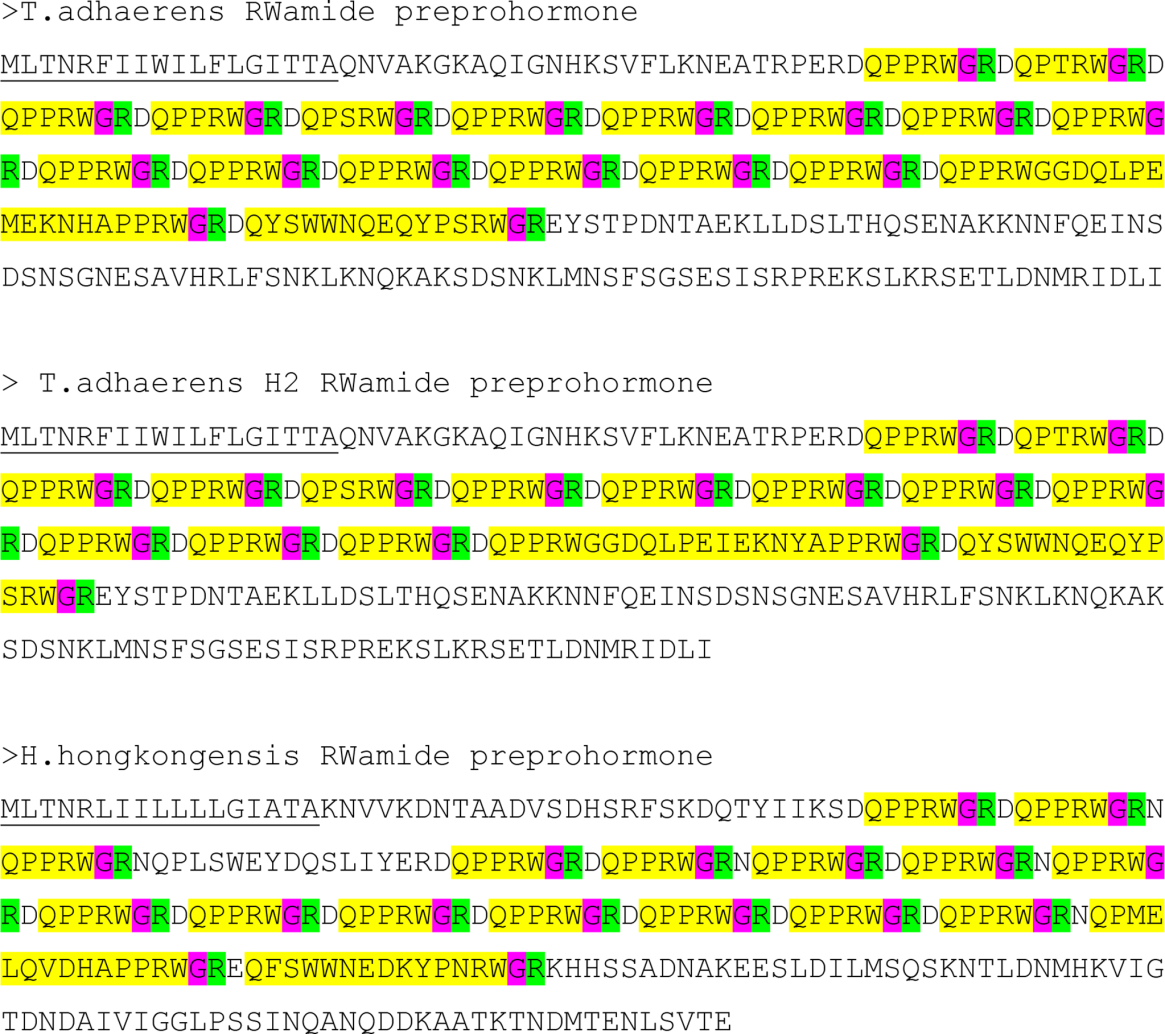
Amino acid residue sequences of three RWamide preprohormones from three placozoan species or lineages: *T*. *adhaerens*, *T*. *adhaerens* sp. H2, and *Hoilungia hongkongensis*. The signal sequences are underlined, the immature neuropeptide sequences are highlighted in yellow, the cleavage sites for prohormone convertase 1/3 are highlighted in green and the C-terminal G residues that are converted into C-terminal amide residues are highlighted in red. Furthermore, each immature neuropeptide sequence contains an N-terminal Q residue that is converted into an N-terminal pQ residue by the enzyme pyroglutaminyl cyclase. Note that each preprohormone is processed into a large number of identical or similar mature neuropeptides, which are protected at their C-termini by amide groups and at their N-termini by pQ groups or pQPP sequences.

In the following, we want to discuss the PRWamide preprohormones as an example of a placozoan peptide preprohormone (**Figure 4**). The PRWamide preprohormone from *T. adhaerens* (strain H1) contains 11 copies of DQPPRWGR and three copies of NQPPRWGR (**Figure 4**). We propose that these immature peptide sequences are likely to be processed into 14 copies of mature pQPPRWamide. It is intriguing that N-terminal peptide processing in Placozoa apparently follows the same principles as N-terminal neuropeptide processing in Cnidaria, as shown by the presence of acidic and other residues (Asp and Asn), but not basic residues, preceding the immature neuropeptides sequences. We find the same prohormone processing in *T. adhaerens* (strain H2), where the PRWamide preprohormone contains 11 copies of DQPPRWamide, which we propose are processed into 11 copies of mature pQPPRWamide. Furthermore, the PRWamide preprohormone from *H. hongkongensis* contains 12 copies of DQPPRWGR and three copies of NQPPRWGR. We propose that these immature peptide sequences will be converted into 15 copies of mature pQPPRWamide. In addition to these pQPPRWamides, the preprohormones from each of the three placozoan species/strains contain a few PRWamide sequences that are slightly different from pQPPRWamide.

The placozoan pQPPRWamide peptide structure reminds us of the LRWamide peptides from Hexacorallia and Ceriantharia (**Supplementary Figure S5**) (76) and of some other peptides with the Wamide C-terminus from other animals (87), but are they really orthologues? We do not believe that this is the case, because: (i) LRWamides have been isolated and sequenced from the hexacoral *A. elegantissima* (41, 42) and have subsequently been annotated from the genomes and transcriptomes of 17 species belonging to the Hexacorallia and Ceriantharia (76). Each species expresses one to four genes, coding for an LRWamide preprohormone and each preprohormone contains one LRWamide peptide. This yields knowledge from altogether 39 LRWamide peptides (**Supplementary Figure S5**). These 39 peptides nearly always have the C-terminal sequence LRWamide and in a few cases IRWamide, or MRWamide (L, I, and M residues are amino acid residues with similar physico-chemical properties) and never PRWamide, or PPRWamide. L and P residues do not have similar properties and P residues disturb the secondary structure of the peptides, making them more rigid. This means that the two peptide families have different secondary structures. (ii) In 38 out of 39 sea anemone LRWamide preprohormones, the LRWamide neuropeptide sequence directly follows the signal sequence (**Supplementary Figure S5**) (76). For the placozoan PRWamide preprohormones, in contrast, there are always long stretches of amino acid residues following the signal sequence, before the first PRWamide neuropeptide sequence appears (**Figure 4**). Besides, there are multiple copies (about 15) of these PRWamide peptides and not only one, as in sea anemones. Thus, both the peptide and the overall preprohormone structures of the LRWamide and PRWamide peptides are essentially different, arguing strongly against the possibility that the two peptide families are orthologous.

For the other placozoan peptide families, we also cannot find orthologues in Cnidaria, or Bilateria (as tested with TBLASTN). In addition, we were unable to discover orthologues, when comparing placozoan GPCRs with GPCRs from Cnidaria (*N. vectensis*), or Bilateria (*H. sapiens*), using extensive phylogenetic tree analyses (**Supplementary Figures S3**, **S4**), except for the LGRs, which we will discuss in Chapter 5. These results suggest a low evolutionary pressure to conserve amino acid sequences in neuropeptides or neuropeptide GPCRs between the three animal groups, which is surprising, because there has been clear neuropeptide conservation, and a clear neuropeptide GPCR conservation in Proto- and Deuterostomia over a period of 700 million years (Chapter 1; **Figures 1**, **2**).

## 5 Leucin rich repeat-containing GPCRs

**Figure 5:**
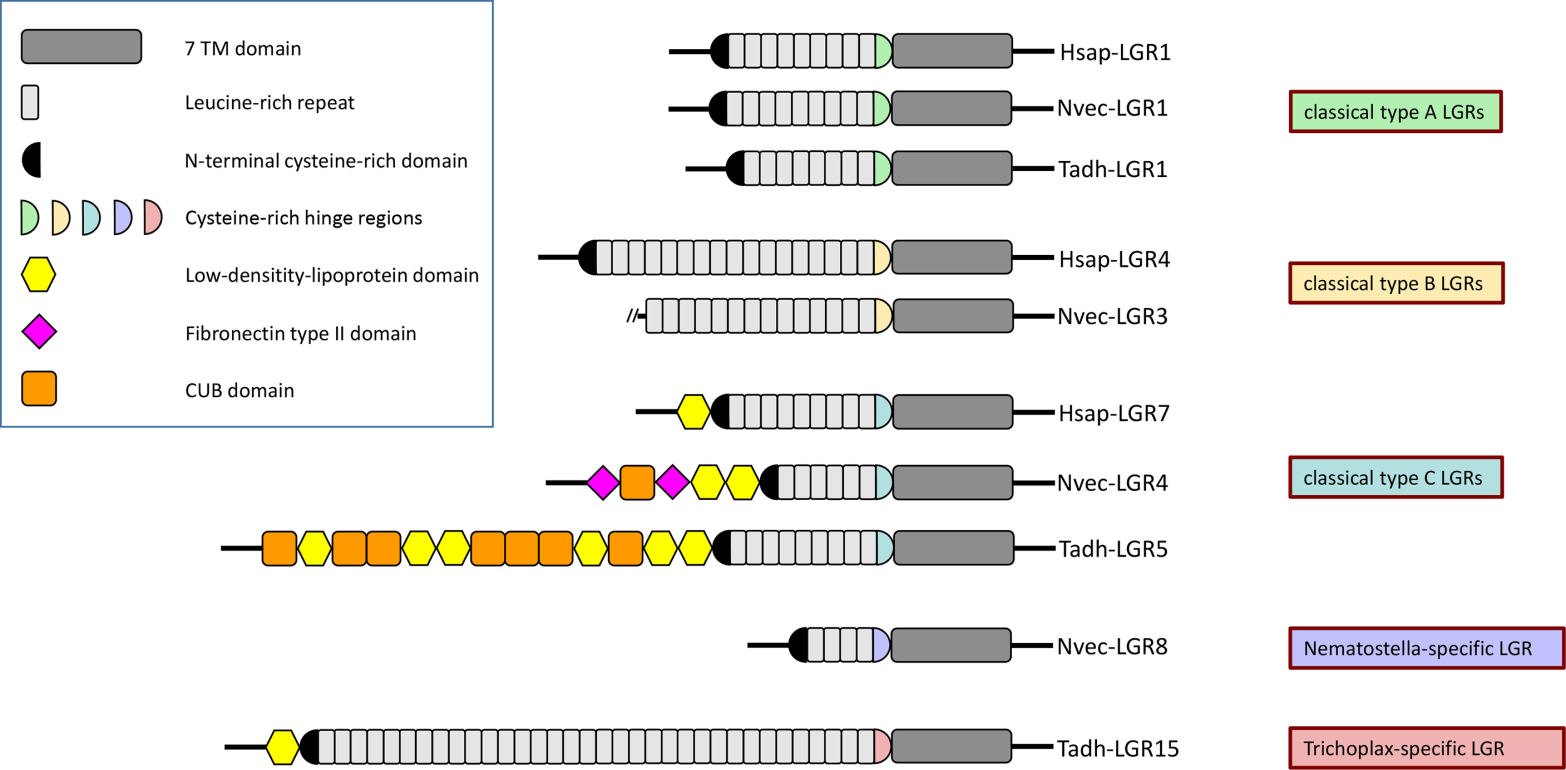
Protein domain composition of the extracellular N-termini of human (Hsap-) LGRs, *N. vectensis* (Nvec-) LGRs, and *T. adhaerens* (Tadh-) LGRs. Humans have three type-A, three type-B, and two type-C LGRs, depending on the structure of the hinge domains connecting the extracellular N-termini to their seven-transmembrane (7TM) regions. These human LGRs are also called LGR-1 to -3, LGR-4 to -6, and LGR-7 to -8, respectively (see main text). The top of this figure shows Hsap-LGR-1 (human FSH receptor), a typical human type-A LGR, containing a type-A-like cysteine-rich hinge region (highlighted in green) that connects the 7TM domain (dark gray) to the 9 leucine-rich repeats (highlighted in light gray) of its N-terminus. The N-terminus starts with another cysteine-rich domain (highlighted in black), preceding the leucine-rich domains. Hsap-LGR-2 and -3 (= human TSH and LH/CG receptors), Nvec-LGR-1 and -2, and Tadh-LGR-1 and -2 have similar overall structures and domains. This figure shows also Hsap-LGR-4, a typical human type-B LGR, containing a type-B-like cysteine-rich hinge region (drawn as a yellow half circle) that connects the 7TM domain to the 16-18 leucine-rich repeats of its N-terminus. Nvec-LGR-3 has a similar type-B-like cysteine-rich hinge region, but its N-terminus is incomplete. Surprisingly, a type-B LGR appears to be lacking in *T. adhaerens* (see **Figure 6**). Furthermore, the figure shows Hsap-LGR-7 (= human relaxin receptor), which is a typical type-C LGR. This receptor has a C-type cysteine-rich hinge region (drawn as a light blue half circle) that connects the 7TM domain with the 9 leucine-rich repeats of the N-terminus. This N-terminus starts with a low-density-lipoprotein (LDL) domain (yellow hexagon) followed by the usual cysteine-rich domains preceding the N-terminal leucine-rich domains. Nvec-LGR4 and Tadh-LGR-5 have similar overall structures including the type C-like hinge regions and the LDL domains at the start of their N-termini. These receptors, however, have also additional N-terminal domains, such as CUB domains (a structural motif of about 110 residues found almost exclusively in extracellular and plasma membrane-associated proteins) (orange boxes), and fibronectin type II domains (pink squares). Finally, *N. vectensis* and *T. adhaerens* have a large number of novel LGRs that do not belong to one of the above-mentioned canonical LGR families. We give here two examples: Nvec-LGR-8, which only contains 4 leucine-rich repeats and that has its own, specific hinge region; and Tadh-LGR-15, which has as much as 34 leucine-rich repeats and, again, its own specific cysteine-rich hinge region (see also **Figure 6**). The accession numbers of the LGR sequences used in this figure can be found in **Supplementary Table S2**.

Leucine rich repeat-containing GPCRs (LGRs) belong to the family-A GPCRs, but distinguish themselves from the other members of this family by the presence of a long N-terminus, containing multiple leucine-rich repeats (**Figure 5**). These leucine-rich repeats are an important part of the ligand binding domains of the receptors (88–90). The ligands of the LGRs are various protein hormones, such as the glycoprotein hormones: Luteinizing hormone (LH), choriogonadotropin (CG), follicle stimulating hormone (FSH), and thyroid stimulated hormone (TSH). LH, CG, FSH, and TSH have one common subunit (the glycoprotein alpha subunit, also called GPA1), while the second subunit of these dimeric hormones is a beta subunit specific for each of the four mentioned hormones (LHbeta, CGbeta, FSHbeta, TSHbeta). These beta subunits are also called GPB1 to GPB4) (91). LH and CG bind to the same receptor, while FSH and TSH have their own specific receptors. There also exist other glycoprotein hormone subunits in humans: Glycoprotein alpha subunit-2 (GPA2), and glycoprotein beta subunit-5 (GPB5) (92). GPA2/GPB5 (also called thyrostimulin) is a dimeric glycoprotein hormone stimulating the TSH receptor, thus acting similarly to TSH (93). Altogether, there are eight LGRs in humans: LGR1 to LGR3 are the FSH, LH/CG, and TSH receptors (in this order); LGR4 to LGR6 are activated by various R-spondin proteins, which signal through the canonical Wnt/beta-catenin pathway (94–97); LGR7 and LGR8 are receptors for relaxins and other insulin-like peptides (88, 98).

LGRs can be classified as type-A, -B or -C, based on the structures of their N-terminal ectodomains and the hinge regions connecting these ectodomains to the transmembrane regions of the LGRs (99) (see also **Figure 5** for details). Type-A and -C LGRs contain about 7-9 leucine-rich repeats (LRRs), whereas type-B LGRs contain about 14-18 LRRs (**Figure 5**). The hinge regions are cysteine-rich and have a subtype-specific consensus sequence with a well-defined number of cysteine residues forming disulfide bridges that stabilize the structure (99). The classification of the LGRs in type-A, -B, and -C, categorizes the human LGRs in the same way as described in the first paragraph of this Chapter (**Figure 5**): Type-A LGRs are the TSH, LH/CG, and FSH receptors; Type-B are LGR4 to LGR6; Type-C are LGR7 and LGR8 (99). This implies (at least in mammals) that each group uses one category of ligands (Type-A: Glycoprotein hormones; Type-B: R-spondins; Type-C: Relaxins and related insulin-like peptides) (99).

In 1997, three years before the sequencing of the *Drosophila* genome, we cloned an LGR from *Drosophila*, which we named *Drosophila* LGR-1 (DLGR1), the first arthropod (and even protostome) LGR to be identified (100). Three years later followed the cloning of a second *Drosophila* LGR, DLGR2 (101). Subsequently the ligands for both LGRs were identified, being *Drosophila* GPA2/GPB5 for DLGR1 (102) and the *Drosophila* glycoprotein hormone bursicon for DLGR2 (103, 104). Bursicon is a heterodimeric glycoprotein and its structure resembles that of human LH, CG, FSH, and TSH. Bursicon promotes darkening (hardening) of the insect exoskeleton after a moult and has several other important roles in insects among them glucose homeostasis (105, 106). *Drosophila* has two other LGRs, Lgr3 and Lgr4, of which *Drosophila* Lgr3 is a receptor for a *Drosophila* insulin-like peptide, Dilp8 (107). These examples from LGRs in Bilateria, therefore, can be added to the impressive list of GPCRs and their ligands that have orthologues in both Proto- and Deuterostomia, showing that these receptor/neurohormone couples must have originated in the common ancestor of these two lineages (**Figure 1**).

**Figure 6:**
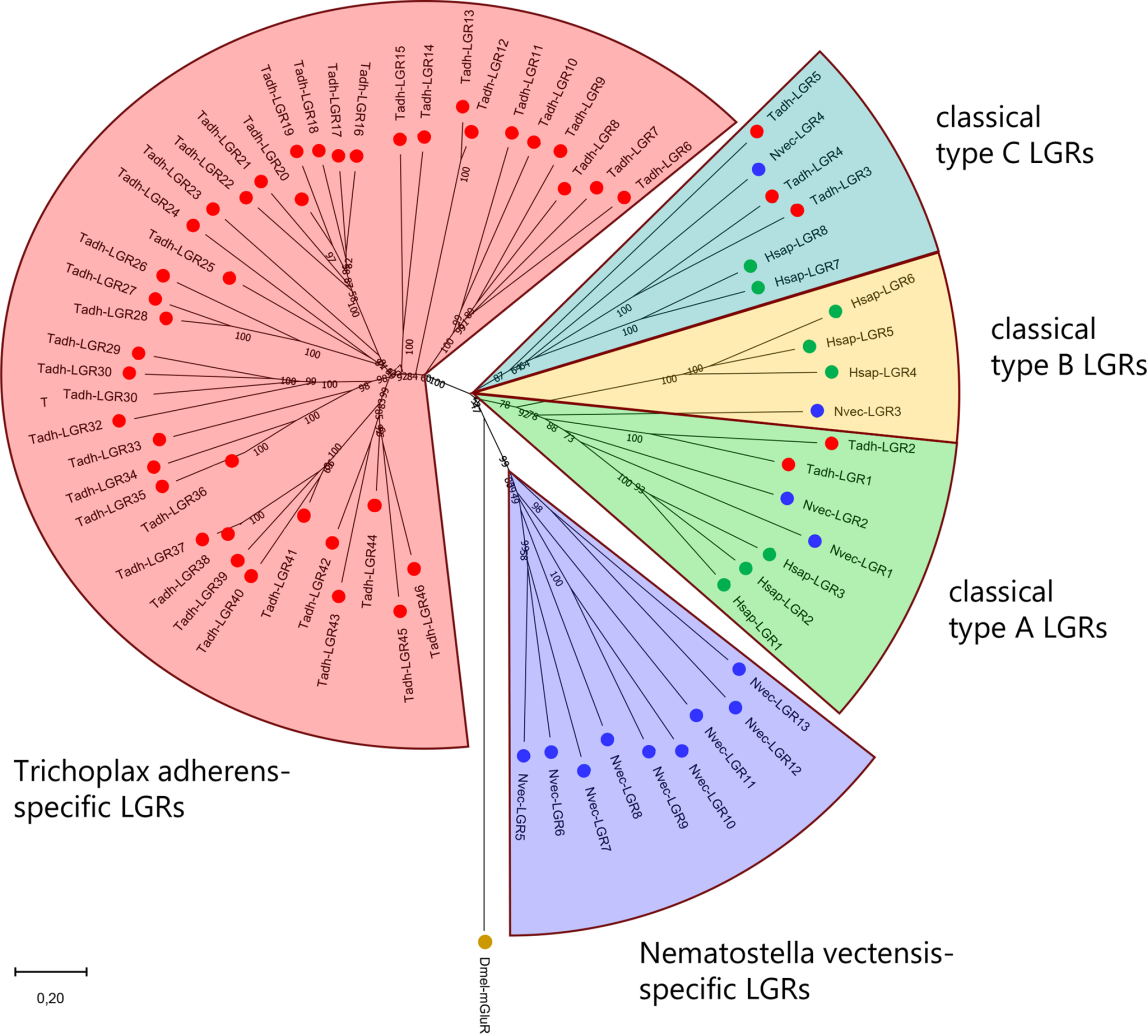
Phylogenetic tree analysis of LGRs from *H. sapiens* (Hsap), the sea anemone *N. vectensis* (Nvec), and the placozoan *T. adherens* (Tadh). Green dots indicate human LGRs, blue dots indicate *N. vectensis* LGRs. Red dots indicate *T. adhaerens* LGRs. Numbers associated with branches of the tree are bootstrap support values. The tree is rooted with the *D. melanogaster* metabotropic glutamate receptor CG11144 (Dm-mGluR). LGRs belong to three families: Type-A (located in the green field of this tree), type-B (yellow field), and type-C (light blue field). Humans have three type-A, three type-B, and two type-C LGRs. *N. vectensis* has two type-A, one type-B, and one type-C LGR. *T. adhaerens* has two type-A, no type-B, and three type-C LGRs. These assignments are independently supported by the presence of specific protein domains in the N-termini of the LGRs (see **Figure 5**). In addition to these three canonical LGR types, *N. vectensis* (purple field) and *T. adhaerens* (red field) have large numbers of novel LGRs that do not belong to the three canonical families: nine in *N*. *vectensis* and forty-one in *T. adhaerens*. The accession numbers of the LGRs used to calculate this tree are provided in **Supplementary Table S2**.

In 1993, when the whole genome sequences from the sea anemone *N. vectensis* or other cnidarians had not been established yet, we were able to clone the first pre-bilaterian LGR from the sea anemone *A. elegantissima* (108–111). Our results from *A. elegantissima* showed, for the first time, that we could follow the evolution of a pre-bilaterian LGR to a bilaterian LGR and that this pre-bilaterian LGR was strongly conserved. We recently created a transcriptome from the cubomedusa *Tripedalia cystophora* and found that this cnidarian contained two LGRs (74). In our current paper, we analysed the genome from the sea anemone *N. vectensis* (110) and discovered that it had thirteen LGRs. After a phylogenetic tree analysis of these sea anemone LGRs and comparing them with the eight human LGRs, we could see that two sea anemone LGRs belonged to type-A, one sea anemone LGR belonged to type-B, and one sea anemone LGR belonged to type-C (**Figure 6**; **Supplementary Table S2**). In addition, there were nine sea anemone LGRs that were neither type-A, -B, or -C. The two sea anemone LGRs from type-A were closely associated with the human TSH, LH/CG, and FSH receptors (**Figure 6**), suggesting that also the sea anemone ligands are dimeric glycoprotein hormones. Similarly, the ligand for the sea anemone LGR from type-B might be an R-spondin; and the ligand for the sea anemone LGR from type-C might be a relaxin or related insulin-like peptide.

Using TBLASTN, we screened the genome from *N. vectensis* with queries corresponding to the alpha and beta subunits of bilaterian glycoprotein hormones and found several candidate ligands for the two type-A sea anemone LGRs (**Supplementary Figure S7**). The next step would be to produce these candidate ligands and test them on mammalian cells transfected with cDNA coding for the sea anemone type-A LGRs. This procedure might be the roadmap for deorphanizing LGRs in pre-bilaterian animals.

There are nine *N. vectensis*-specific LGRs (located in the purple field of **Figure 6**) that in a phylogenetic tree cannot be associated with bilaterian LGRs. Therefore, we cannot predict the ligand classes for them. These LGRs have a lower number of leucine rich repeats in their N-termini and different hinge regions compared the LGRs from group-A, -B, and -C (**Figure 5**). We do not understand why sea anemones would need these nine additional LGRs, but LGRs in sea anemones have apparently a broad range of actions.

We also analysed the LGRs from Placozoa. Nikitin and Roch et al. were the first to discover two LGRs in the genome from *T. adhaerens* (86, 112). We revisited these annotations, using updated genomic and transcriptomic databases from *T. adhaerens* (85), and found no less than forty-six placozoan LGRs (**Figure 6**) (77). Among them were two type-A LGRs, no type-B, and three type-C LGRs. In addition to these five LGRs, there were forty-one LGRs that were specific for *T. adhaerens* (located in the red field of **Figure 6**) (77). These *T. adhaerens*-specific LGRs had hinge regions that were different from the placozoan type-A and type-C LGRs and also had an unusually high number of leucine-rich repeats in their N-termini (**Figure 5**). The presence of forty-six placozoan LGRs is surprising for such a primitive animal like *T. adhaerens*, which only has a simple behavioral repertoire (113).

In conclusion, LGRs have a long evolutionary history, going all the way back to the common ancestor of Cnidaria and Placozoa, which is far beyond 700 MYA (**Figure 1**). Furthermore, due to high amino acid sequence conservations, it is possible to follow the evolutionary “jump” of LGRs from pre-bilaterian animals to bilaterians, something that has not been possible, so far, with the neuropeptide GPCRs or their ligands. During evolution, both the LGRs (**Figure 6**), and their potential ligands (**Supplementary Figure S7**) have been well conserved.

Roch and Sherwood (112) described one or two genes for an LGR and a potential glycoprotein hormone candidate in genomic databases from Ctenophora and Porifera, which are the other two early-branching animal phyla that diverged before the emergence of Bilateria. Thus, the LGRs and their ligands were apparently broadly present in the early metazoans that lived on Earth before the emergence of the Bilateria.

## 6 Neuropeptide-gated ion channels

Peptidergic signaling through GPCRs is slow, due to the use of many steps involved in second messenger cascades. For a few neuropeptides, however, peptidergic signalling can be fast, namely for those peptides that directly gate ion channels, which leads to fast postsynaptic depolarizations. The ion channels involved belong to a small subfamily of the degenerin/epithelial Na^+^ channel (DEG/ENaC) protein family that are channels for Na^+^ ions. To this DEG/ENaC family also belong other physiologically important channel proteins, such as the acid-sensitive ion channels (ASICs). DEG/ENaC proteins consist of three subunits. Each subunit crosses the cell membrane twice and has a large extracellular loop (114, 115). Neuropeptide-gated Na^+^ channels were first discovered by Cottrell and co-workers in 1990 during patch-clamp experiments, where molluscan FMRFamide was applied to neuronal outside-out cell membrane patches from the snail *Helix aspersa* (116). This FMRFamide-gated Na^+^ channel was subsequently cloned by Lingueglia and colleagues in 1995, showing that it was a member of the DEG/ENaC family, and afterwards functionally expressed in frog oocytes, showing that it was a homotrimer (117).

In the following years, FMRFamide-gated ion channels were discovered in a number of molluscs, belonging to different molluscan classes, among them the gastropods *Lymnaea stagnalis* (118), *Aplysia kurodai* (119), and *Lottia gigantea* (120), the bivalve *Crassostrea gigas* (120), and the cephalopod *Octopus bimaculoides* (120). Already in the beginning of these studies, these FMRFamide-gated DEG/ENaC proteins were named FaNaCs (118).

In 2007 Gründer and colleagues found that the cnidarian *Hydra magnipapillata* also contained ion channels acting like the molluscan FMRFamide-gated channels and that these cnidarian channels were gated by two *Hydra* neuropeptides: Hydra-RFamide-1 (pQWLGGRFamide) and Hydra-RFamide-2 (pQWFNGRFamide) (121). These channels were, therefore, called HyNaCs (121). Subsequent studies showed that the HyNaCs were heterotrimers, consisting of combinations of three out of ten different HyNaC subunits that were expressed in *Hydra* and that some of these trimer combinations not only had Na^+^ permeability, but also high Ca^2+^ permeability. The HyNaCs were, in fact, the first members of the DEG/ENaC family to be discovered with high Ca^2+^ permeability (121–124).

Several phylogenetic trees of the HyNaCs, FaNaCs and other members of the DEG/ENaC family from molluscs, nematodes, arthropods and chordates can be found in (125). These cladograms, using both Bayesian and maximum likelihood analyses, show that the HyNaC subunits are not closely related to the FaNaCs, because they are sorted into two clearly different branches of the DEG/ENaC family tree with the HyNaCs grouped together with the ASICs, and the FaNaCs grouped together with the DEGs and ENaCs (125). This is an important finding, as it reveals that the *Hydra* and the snail neuropeptide-gated channels are not close orthologues. In Chapter 3, we have explained that we cannot conclude that cnidarian neuropeptides (for example the Hydra-RFamides-1 and -2) are evolutionary related to RFamide neuropeptides from bilaterians (for example FMRFamide). The presence of neuropeptide-gated ion channels in cnidarians and molluscs, therefore, cannot necessarily be regarded as the evolutionary conservation of a neuropeptide/receptor pair from pre-bilaterians to bilaterians. Of course, several members of the DEG/ENaC family can be gated by neuropeptides and this property has been conserved from pre-bilaterians to bilaterians. The peptide-gated family members in cnidarians, however, might not be directly related to one of the peptide-gated family members that we find in bilaterians today.

Although members of the DEG/ENaC family are probably present in all bilaterians, neuropeptide-gated DEG/ENaC family members have not been found in deuterostomes. In protostomes, however, in addition to the earlier-mentioned FaNaCs from molluscs, also the annelid *Platynereis dumerilii* expresses peptide gated ion channels. These channels, however, are not gated by FMRFamide or RFamide peptides, but by annelid-specific peptides having the C-terminal Wamide residue, such as GWKQGASYSWamide (= myoinhibitory peptide-2). They were, therefore, named myoinhibitory peptide-gated ion channels (MGICs) (126) and recently re-named as Wamide-gated Na^+^ channels (WaNaCs) (120). In various phylogenetic tree analyses, the molluscan FaNaCs and the annelid WaNaCs turned out to be orthologues, while the cnidarian HyNaC subunits were not orthologous to the molluscan/annelid subunits (120, 126). These results confirm our earlier suggestion that the cnidarian HyNaCs might not have direct evolutionary relationships to the peptide-gated ion channels from bilaterians.

In a recent paper, Dandamudi and co-workers tested a large number of molluscs, annelids, platyhelminths and other lophotrochozoans (**Figure 1**) for the presence of FaNaCs and WaNaCs (120). The results from that work showed that FaNaCs were present in the majority of lophotrochozoans, but also that a small group of annelids, among them *P. dumerilii*, had WaNaCs instead of FaNaCs.

No reports have been published about the presence of peptide-gated DEG/ENaC channels in Ecdysozoa (**Figure 1**) and, as mentioned earlier, in Deuterostomia. It seems, therefore, that these peptide-gated DEG/ENaCs are limited to two animal clades: To the larger clade of lophotrochozoans and the smaller clade of cnidarians. It is possible, however, that peptide-gated ion channels also occur in placozoans, because references (77, 125) independently describe the presence of around ten DEG/ENaC subunits in the placozoans *T. adhaerens*, and *H*. *hongkongensis*. In phylogenetic tree analyses, the placozoan subunits lie on the same branch as HyNaCs and ASICs, and far away from the FaNaCs, suggesting that they might have properties similar to HyNaCs (77, 125). However, both publications describe the presence of one placozoan subunit that lies on the same branch as the FaNaCs, suggesting that placozoans might have two types of peptide-gated DEG/ENaCs. These possibilities based on phylogenetic tree analyses, however, need to be validated by laboratory experiments.

## 7 Endocrine cells but no neurons in Placozoa

**Figure 7:**
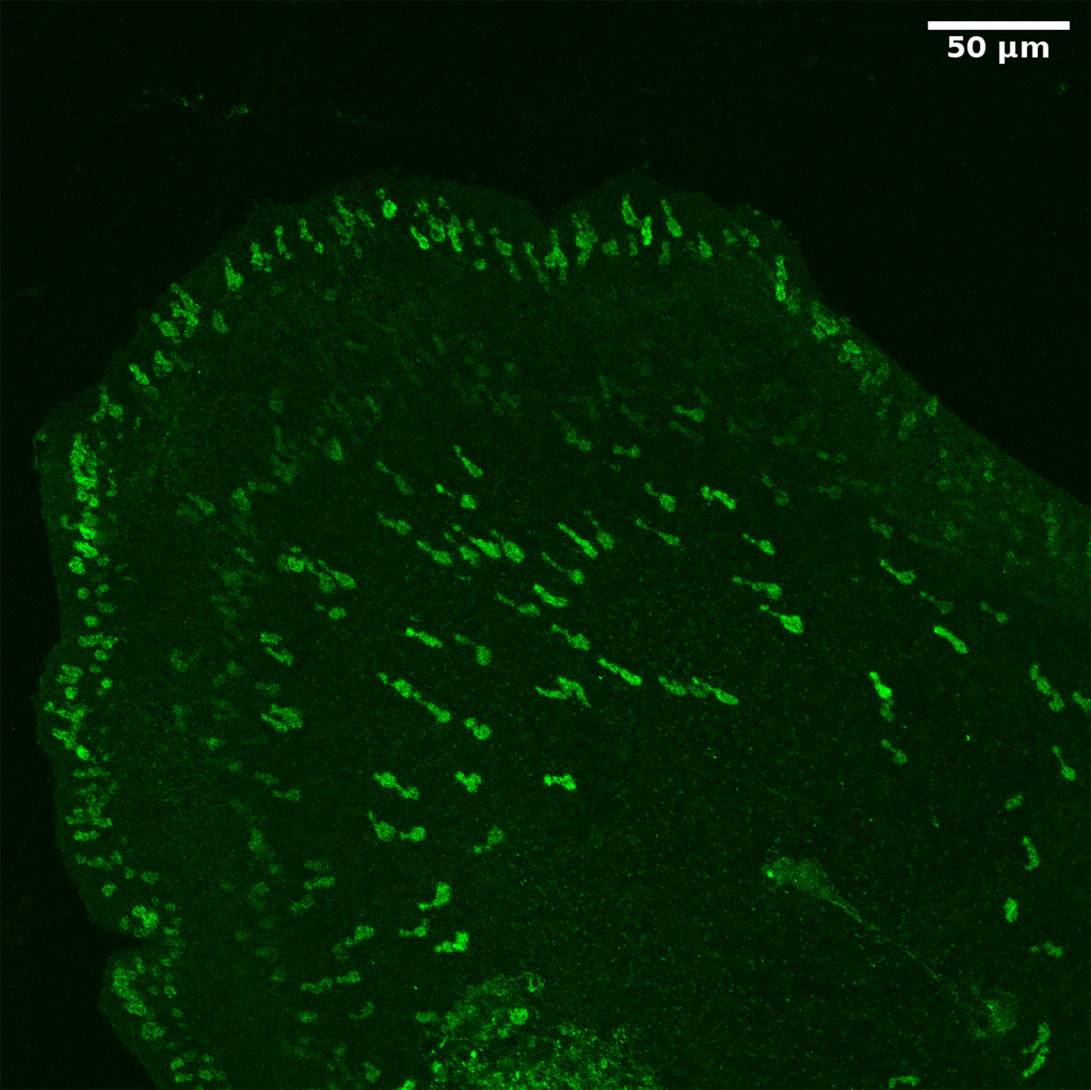
Whole mount staining of *T. adhaerens*, using an antiserum against the neuropeptide processing enzyme glutaminyl cyclase from *T. adhaerens.* We can observe staining of a large number of endocrine cells. These cells are flask-shaped and have protrusions towards the surface of the animal (please, see its margin), where they probably receive sensory input.

For long, it had been uncertain whether placozoans had a nervous system. We answered this question by raising antibodies against the various predicted placozoan neuropeptide sequences and applying these antibodies to fixed placozoans during whole mount immunocytochemistry (77). However, already in the beginning of our work, we were “surpassed” by a publication from Varoqueaux and colleagues (113), who showed that all known neuropeptides in placozoans were produced by endocrine cells. We reached the same conclusions when we stained *T. adhaerens* with our own neuropeptide antibodies (77). Many neuropeptides in *T. adhaerens* have an N-terminal pyroglutamate residue. Therefore, we also raised antibodies against the processing enzyme pyroglutaminyl cyclase from *T. adhaerens*, which we annotated from its genome sequence (77). **Figure 7** shows the staining of *T. adhaerens* by one such antibody. Again, only endocrine cells could be stained (77).

In mammals, many neuropeptides are produced by both nerve cells and endocrine cells. A classical example of this phenomenon is somatostatin, which was one of the first neuropeptides to be isolated from mammalian hypothalami in the 1960s and 1970s (127, 128). Ten years later, when immunocytochemistry had been introduced as an extremely versatile cell staining technique, Dubois found that somatostatin was not only produced by neurons from the hypothalamus, but also by endocrine cells from the stomach, the so-called D-cells (129). Somatostatin released from the D-cells inhibits the release of gastrointestinal hormones by other endocrine cells in the stomach, thereby blocking gastric acid secretion (130).

Also in Protostomia, like *D. melanogaster*, there are several neuropeptides that are produced in enteroendocrine cells of the gut, such as diuretic hormone-31, the allatostatins-A, -B, and -C, CCHamides, neuropeptide-F, and tachykinins (131–134). These peptides, however, like the other neuropeptides from *Drosophila*, are also expressed in neurons of the central nervous system (135). Both in protostomes and deuterostomes, therefore, neuropeptide genes may be expressed in neurons and endocrine cells.

How is the situation in Cnidaria? Yearlong ultrastructural work by Jane Westfall on cnidarians has shown that two types of neurons exist in cnidarians: “sensory cells” and “ganglion cells” (136). Cnidarians have two cell layers, ectoderm and endoderm, which are separated by a layer of extracellular matrix, called the mesoglea. Sensory cells are part of either the ectoderm or endoderm, and are orientated perpendicularly to the mesoglea. These cells have protrusions towards the apical parts of their cell layer and are typically bearing a sensory cilium that reaches out into the gastric cavity of the animal, or into the surrounding seawater (136). The ganglion cells are lying in the basal portions of the two cell layers and are a part of in the nerve net of the animal. Ganglion cells also connect to the sensory cells *via* synapses. Both sensory cells and ganglion cells have long processes and form multiple synapses, so they have the properties of genuine neurons (136).

As mentioned in Chapter 3, cnidarian nervous systems are strongly peptidergic (34–71, 74–77). By using antibodies against the different cnidarian neuropeptides, therefore, it is possible to stain the various populations of nerve cells in high detail. Such staining shows both sensory cells and ganglion cells connected to densely or loosely woven nerve nets or giant nerve fibres (35–37, 56–61). We have never realized that among the stained cells also might be endocrine cells, simply because their cell shapes make them look like sensory cells. Yet, we were sometimes wondering about scattered single immunostained sensory cells with no processes that were located in the endoderm of anthozoans, for example in the endodermal ovaries of octocorals (60). An example of these cells can be seen in **Supplementary Figure S8**. Therefore, it did not come as a complete surprise to us, when a publication appeared this year, showing that the anthozoan *N. vectensis* had genuine endocrine cells (137). These cells became visible in transgenic *N. vectensis* embryos, expressing the transcription factor *N. vectensis* insulinoma-associated protein 1 (*Nv*Insml-1) coupled to green fluorescent protein (GFP). In vertebrates, *insml-1* expression is required for the development of central and peripheral neurons and for stem cells that are committed to differentiate into endocrine cells (138). The orthologous gene in *N. vectensis*, *NvInsml-1*, appears to have similar properties. The gastrula stage was the first stage in which *N. vectensis* embryos, transgenically expressing the *NvInsml-1-GFP* gene, showed labelled cells, which anatomically looked like endocrine cells. These presumed endocrine cells had the appearance of sensory cells with protrusions towards the apical part of the ectoderm and an apical cilium, but lacking their basal nerve processes (dendrites) (137). In their planula stages, the transgenic embryos expressed GFP in typical sensory cells, and in their primary polyp stages, these transgenic lines expressed GFP in ganglion cells. Cnidarians, therefore, appear to have (i) endocrine cells (ii) sensory cells, and (iii) ganglion cells, while placozoans only have endocrine cells (113). The situation in cnidarians resembles that of mammals and other bilaterians, while placozoans are unique for only having endocrine cells that are responsible for peptidergic signaling.

Cnidaria and Placozoa probably form a monophyletic clade based on their common ways of prohormone processing, which is somewhat different from that in Bilateria (Chapter 3, Chapter 4). Also, molecular phylogenetic work from other laboratories supports a single clade of Cnidaria and Placozoa (139). This brings up the question of whether the common ancestor of cnidarians and placozoans had (i) endocrine cells (like in placozoans); or (ii) endocrine cells, sensory cells, and ganglion cells (like in cnidarians). Of course, this question is hard to answer in an experimental way. The above-mentioned experiments with transgenic *N. vectensis* embryo’s (137), however, very neatly show a sequential appearance, during sea anemone embryonic development, of first endocrine cells (in gastrula stages), then sensory cells (in planula stages), and finally ganglion cells (in primary polyps). If one could accept that essential parts of Ernst Haeckel’s “Biogenetic Law” (“ontogeny recapitulates phylogeny”) is still valid in modern biology (140, 141), this consecutive appearance of the three cell types would support the idea that endocrine cells evolved first.

## 8 Concluding remarks

Neuropeptides and their GPCRs are present in bilaterians, cnidarians, placozoans, and possibly other animal taxa. In placozoans, it would be more appropriate to call these peptides “endocrine peptides”, because nerve cells are apparently lacking in this early-branching animal phylum. It is hard to see evolutionary relationships between the neuropeptide/GPCR couples across bilaterians, cnidarians, and placozoans, although within bilaterians, these relationships between proto- and deuterostomes are obvious. Yet, a special group of neuropeptide-related hormones, the glycoprotein hormones and their GPCRs, the LGRs, are considerably conserved over a long evolutionary period. Especially in the early-branching phyla, the LGRs have strongly radiated, which culminated in placozoans having forty-six LGRs compared to eight in humans.

Genomics, transcriptomics, and bioinformatics have been invaluable tools for our understanding of cnidarian and placozoan neuro-endocrine evolution (34, 74–76). These techniques have already transformed large areas of biology today and will certainly continue to do so in the near future.

## 9 Materials and methods

For **Supplementary Figure S3**, the human family-A GPCRs, excluding the olfactory receptors, were downloaded from GPCR-PEn (http://gpcr.utep.edu). Cnidarian family-A GPCRs, predicted from the sequenced genome of *Nematostella vectensis*, were downloaded from uniprot (http://uniprot.org). Placozoan family-A GPCRs, annotated from the re-annotated genome and the predicted proteome of *T.adhaerens*, were downloaded from genomeevolution.org (142). The receptors were extracted using the HMMER hmmsearch method using the EMBL-EBI Pfam rhodopsin GPCR model (pfam.xfam.org/). Sequences with at least 5 predicted transmembrane domains, as predicted by TMHMM, were kept for analysis.

The receptors were aligned using MAFFT v7.487, using the FFT-NS-2 alignment strategy. The alignments were trimmed using trimAl v1.4 with the -gt 0.1 settings. The trees were constructed using IQ-Tree v1.6.12 with the JTT model on a single thread. Bootstrap values were calculated using IQ-Tree’s UFboot method with 1000 replicates.

For the phylogenetic tree analyses of **Figures 2**, **6**, the sequences were aligned by ClustalW using MEGA v11.0.11, but their N-termini were not trimmed. The accession numbers of the proteins used for **Figure 2** are shown in **Supplementary Table S1**. The accession numbers of the proteins used for **Figure 6** are shown in **Supplementary Table S2**. The trees were constructed with the neighbor-joining method. Bootstrap values were calculated with 1000 replicates.

## Author’s note

This paper is part of the research topic “Genomic and Transcriptomic Insights into Neuroendocrine Evolution and Function”, hosted by Drs Dan Larhammar, Isabel Beets, Maurice Richard Elphick, and Elizabeth Amy Williams.

## Author contributions

FH, TK, and CG conceived and designed the project and analyzed the data. CG wrote the paper with continuous input from the other authors. All authors approved the final manuscript.

## Funding

We thank the Danish Council for Independent Research (grant number 7014-000088 to CG) for financial support. This funding body played no role in the design of the study and collection, analysis, and interpretation of data and in writing the manuscript.

## Conflict of interest

The authors declare that the research was conducted in the absence of any commercial or financial relationships that could be construed as a potential conflict of interest.

## Publisher’s note

All claims expressed in this article are solely those of the authors and do not necessarily represent those of their affiliated organizations, or those of the publisher, the editors and the reviewers. Any product that may be evaluated in this article, or claim that may be made by its manufacturer, is not guaranteed or endorsed by the publisher.
