# Supplementary material for: Review: The evolution of peptidergic signaling in Cnidaria and Placozoa, including a comparison with Bilateria
Source: Front Endocrinol (Lausanne). 2022 Sep 23;13:973862. doi: 10.3389/fendo.2022.973862 (PMC9545775; doi:10.3389/fendo.2022.973862)
Supplement: Supplementary file 1 [file Image_1.pdf]

Supplementary Figure 1

Comparison of cnidarian with bilaterian RFamides. **(A)** Cnidarian RFamides (upper five lines, names highlighted in light green) are compared with RFamides from protostomia (light blue) and deuterostomia (light red). The sequences of the most common mature peptides from each precursor are given in the right column. A phylogenetic tree calculated with these sequences using MEGA 11 is shown in the left column. While the tree was used for sorting, it cannot be used for establishing phylogenetic relationships, because the bootstrap values are too low. Yellow boxes mark the N-terminal signal peptides, blue boxes the peptide copies on the precursors. The scale bar at the top indicates the length of the precursors in amino acids. **(B)** Phylogenetic tree analysis of the complete RFamide precursor sequences, calculated with the program CLC Main Workbench 6.0. Precursor names and accession numbers: Aele-RFa, *Anthopleura elegantissima* antho-RFamide (AAA27738); Nvec-RFa, *Nematostella vectensis* antho-RFamide (XP_048577320); Hmag-RFa1, *Hydra magnipapillata* RFamide 1 or preprohormone A (Y11678); Hmag-RFa2, *H. magnapapillata* RFamide 2 or preprohormone B (Y11679); Hmag-RFa3, *H. magnapapillata* RFamide 3 or preprohormone C (Y11680); Lsta-FMRFa2, *Lymnea stagnalis* FMRFamide 2 (P42565); Bflo-FMRFa, *Branchiostoma floridae* FMRFamide (XP_035657985); Ttra-FMRFa, *Terebratalia transversa* FMRFamide (ASW15421); Cele-flp1, *Caenorhabditis elegans* flp1 (NP_501592); Lsta-FMRFa1, *L. stagnalis* FMRFamide 1 (P19802); Dmel-FMRFa, *Drosophila melanogaster* FMRFamide (NP_523669); Cele-flp3, *C. elegans* flp3 (NP_509694); Dmel-SK, *D. melanogaster* drosulfakinin (NP_524845); Lsta-LFRFa, *L. stagnalis* LFRFamide (AAV41057); Hsap-QRFP, *Homo sapiens* pyroglutamylated RFamide (AAI01129); Hsap-Kisspeptin, *H. sapiens* Kisspeptin (NP_002247); Dmel-sNPF, *D. melanogaster* short neuropeptide F (Q9VIQ0); Hsap-NPY, *H. sapiens* neuropeptide Y (); Hsap-NPFF, *H. sapiens* neuropeptide FF (NP_003708); Hsap-NPVF, *H. sapiens* neuropeptide VF or gonadotropin-inhibitory hormone (NP_071433).

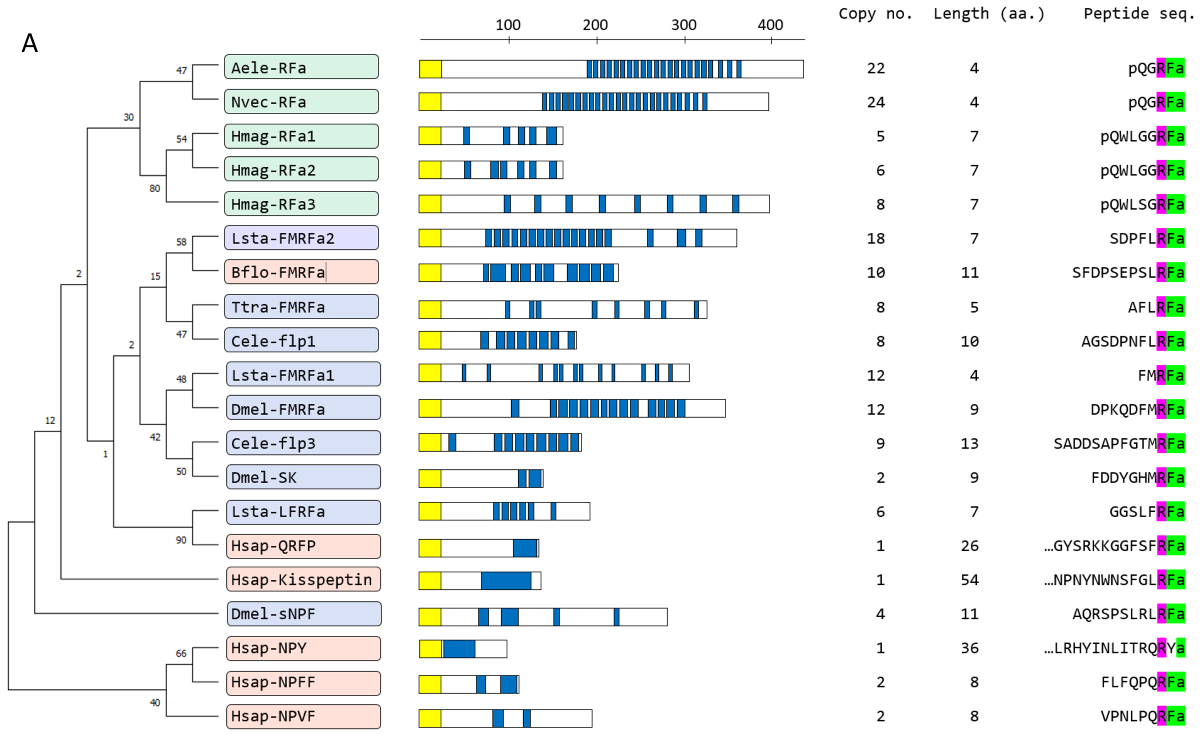

B

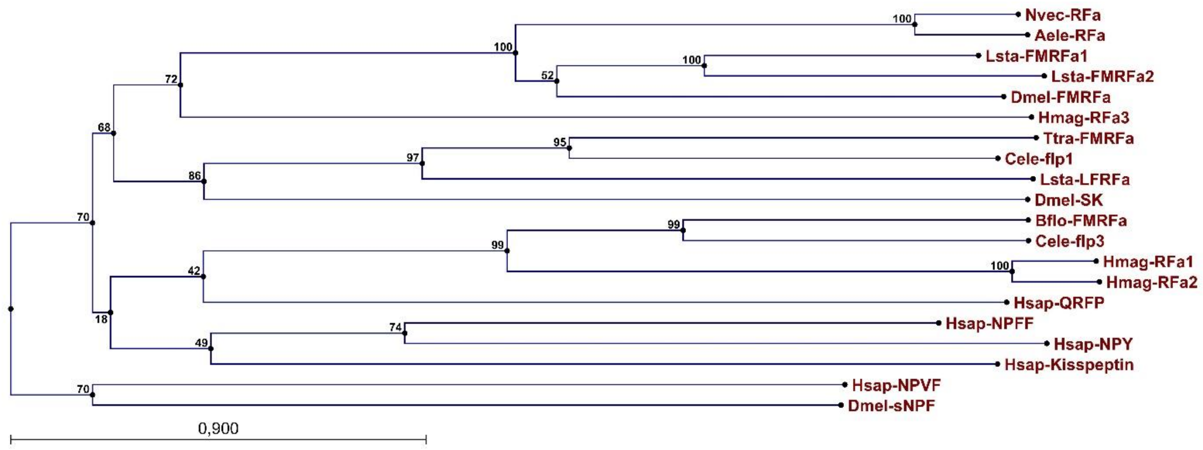

Nvec-RFa
Aele-RFa
Lsta-FMRFa1
Lsta-FMRFa2
Dmel-FMRFa
Hmag-RFa3
Ttra-FMRFa
Cele-flp1
Lsta-LFRFa
Dmel-SK
Bflo-FMRFa
Cele-flp3
Hmag-RFa1
Hmag-RFa2
Hsap-QRFP
Hsap-NPFF
Hsap-NPY
Hsap-Kisspeptin
Hsap-NPVF
Dmel-sNPF
100
100
100
52
72
68
95
97
86
70
99
99
100
42
18
74
49
70
0,900
