# Supplementary material for: Review: The evolution of peptidergic signaling in Cnidaria and Placozoa, including a comparison with Bilateria
Source: Front Endocrinol (Lausanne). 2022 Sep 23;13:973862. doi: 10.3389/fendo.2022.973862 (PMC9545775; doi:10.3389/fendo.2022.973862)

Supplementary Figure 3

Detailed view of some branches of the phylogenetic tree (see Additional file 2) in linear orientation. Bootstrap values are given below the branches. In addition, the names of the human GPCRs are shown. There are two orthologues for type-A LGRs in both *Nematostella* and *Trichoplax* and one orthologue for family B LGRs (LGR-4, -5 and -6) in Nematostella. Furthermore, there are neuropeptide receptor candidates in *Trichoplax* clustering with either the human GnRH receptor or NPS receptor. In *Nematostella*, there is one GPCR showing similarity to the human prolactin releasing peptide receptor (PrRP-R) and one similar to the bradykinin receptors BK-R1 and BK-R2. In both *Trichoplax* and *Nematostella*, we identified one receptor with similarity to human serotonin receptors. The *Trichoplax* candidate shows also some similarity to human trace amine receptors (TAARs). We want to stress, that these orthologous relationships between the human and Nematostella and Trichoplax GPCRs have to be supported by molecular cloning and subsequent deorphanization of the invertebrate receptors.

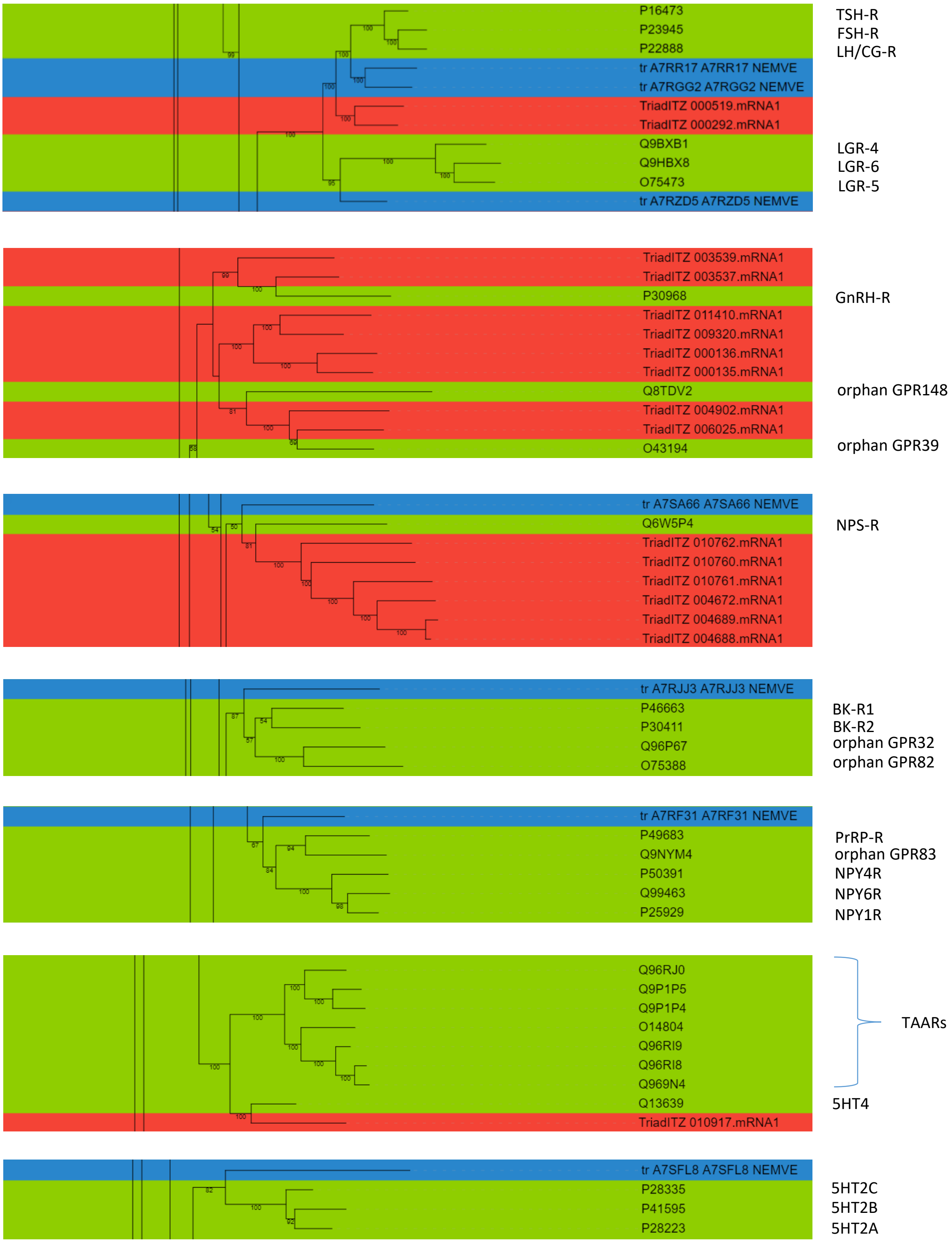

Supplement: Supplementary file 3 [file Image_3.pdf]
