# Supplementary material for: Review: The evolution of peptidergic signaling in Cnidaria and Placozoa, including a comparison with Bilateria
Source: Front Endocrinol (Lausanne). 2022 Sep 23;13:973862. doi: 10.3389/fendo.2022.973862 (PMC9545775; doi:10.3389/fendo.2022.973862)
Supplement: Supplementary file 4 [file Image_4.pdf]

Supplementary Figure 4.

This file is identical to "Additional file #10" from ref. [75]

Amino acid sequences of the Antho-RWamide or related preprohormones in species belonging to the cnidarian subclasses Hexacorallia and Ceriantharia. Antho-RWamide-1 (pQSLRWamide) and Antho-RWamide-2 (pQGLRWamide) have been isolated and sequenced from Anthopleura elegantissima. Signal sequences are underlined. An asterisk indicates a stop codon. Neuropeptide sequences are highlighted in yellow; C-terminal processing sites are highlighted in green. The C-terminal Gly residues that are converted into C-terminal amide groups are highlighted in red.

Actiniaria (see Tables 3, 5, 9; neuropeptide family 10 from ref [75])

Anthopleura elegantissima

>GBXJ01064083.1 TSA: Anthopleura elegantissima comp58479_c0_seq2 transcribed RNA sequence

MASKTYLITLLVSCLLISVCIQHSNAQQQGLRWGKKSVDSMEQQINEEKAADELRRFKDYFKRKYNQDAIF*

>GBXJ01137852.1 TSA: Anthopleura elegantissima comp71170_c0_seq1 transcribed RNA sequence

MNQARLFVFLALTCLVVLTHVSDTKAQGLRWGRTMQDESNGIMDERGLPNERNSGKYWDDIFPQRVLRKRFLA
KKNADK*

>GBXJ01137879.1 TSA: Anthopleura elegantissima comp71197_c0_seq1 transcribed RNA sequence

MESKKLLAILVISCVLVTLCFQTTEAQSLRWGREFDDKYPEARNMKEFLQRDANNKKREYKFNRS*

>GBXJ01137947.1 TSA: Anthopleura elegantissima comp71268_c0_seq1 transcribed RNA sequence

MDAKKIIIFAVISCVLLSVCIDETSAQGGLRWGREYEAANQRESPPEGWPEMKRNFLKKRAFKFNDAA*

Anemonia viridis

>GHCD01087696.1selectionselectionrevtranslationframe+1

MESKKLLAILVISCVLVTLCFQTTEAQSLRWGREFEDKYPEARNIKEFLQRDAKNKKREYNYNRS*

>GHCD01033550.1selectionselectiontranslationframe+1

MNAKKIIIFAVISCVLLSVCIDETSAQGALRWGREYEAMNQKESPPAEGWSEMKRNFLKKRAFKFNDAA*

>GHCD01073236.1selectionselectionrevtranslationframe+1

MASKTYLITLLVSCLLVSMCIHQSNGQQQQGLRWGKKSVDSMEQEINEEKAADELRRFKDYFKRKYNQDAIF*

Nematostella vectensis

>HADP01262889.1selectionselectiontranslationframe+1

MASKTLLVCLLVTFMVLSIYTQESSAGPPQGLRWGKRWENPSEKQVRENAEREVQDFKDYFKKKYNRDLDI*

>HADP01080760.1selectionselectiontranslationframe+1

MESKRLLIVLVVCCALVSLYVEPSQAQGLRWGREFEEEHPKLSPLMKEYLRRQEAMQKKREFAAKKADY*

>HADP01056499.1selectionselectionrevtranslationframe+1

MESKRLLVILVVCFALVAFAPSTEAQGLRWGREYDESPEKLVPMMKEYIRRQEELKKKREFSMN*

>HADP01189706.1selectionselectiontranslationframe+1

MDAKKVLLIAVISCVLLSVCIEQTTAQGGLRWGREFENEFQQWKDAHYPSREERRSFQKKRSFKMA*

Phymanthus crucifer

>WUCR01012252.1selectionselectiontranslationframe+1

MASKTYLISLLVCCLLISVCIQQSNAQKPPGLRWGKKSVDSMEEQINEDKAADELRRFKNYFKRKYNRGR*

>WUCR01013596.1selectionselectiontranslationframe-1

MRQARFFVLLAVSCLVVLSFVSYSEAQGIRWGKPKQNASNSIVDERGSPNERNSGNYWDDIIPQRAMRKRFLS
KKTAGRMFD*

Scolanthus callimorphus

>GGGE01325634.1selectiontranslationframe+1

MDSKRLLVVLVICCALVSLYVEPTEAQGLRWGREYEDEMPKLRELMKEYIKNQAKKRREFAMKEKL*

>GGGE01295518.1selectiontranslationframe-1

MDSKRLLVVLVLCCALVSLYVEPTEAQGLRWGKRDADDEMANLKEFMKEYMMRNQAKKRREFSGH*

>GGGE01326337.1selectiontranslationframe+2

MDARKFLVLAVIACVLVSVCVEQTSAQGGLRWGKRNFVNYIEEMLSRLSPAEKRSLLKRTLNANDE*

>GGGE01334503.1selectiontranslationframe-2

MASKTLLVCLLISFMVISLYTEQTSAQSQGLRWGKKSVDSELEEDQKAEAELRKFRQYFKRKYHRDFAY*

Exaiptasia diaphana

>TSA: Aiptasia pallida Loc_18862_Tr_1 mRNA sequence

MESKKLMILLVISCVLLSVSVDFTNAQGLRWGRREFNDDYADAKAFKEWLEQRDANKKREYKANRS*

>TSA: Aiptasia pallida Loc_47792_Tr_1 mRNA sequence

MDTKKIILFAVFACILLSVCVEESCAQMHLRWGRELEDQDRDALLKWIWNKRSAQKNKKFKSNGWE*

Scleractinia (see Table 5, neuropeptide family 10)

Acropora millepora

>GHGQ01064576.1selectiontranslationframe+2

METKNLVAVLFVSCIFLSICLQPTASQGLRWGREFEEEHPRWKTVKSDYRRKNLHERKFDTSAEKAFDFGRH*

>GHGH01047445.1

MVSSNKLVLLCLIFGLLLSTLSRPAGGQLLGIRWGRNYQDNDVNREVHKPKLWESMTERKFSPEIVQGRQAGR
VLKKLLHERQRDKLDNQ*

Acropora digitifera

>NW_015441410.1selectionselectionrevtranslationframe+1

METKNLVAVLFVSCIFLSICLQPTASQGLRWGREFEEEHPRWKTVKSDYRRKNLHERKFDTSAEKGKDTILCY
M*

Mantipora capitata

>RDEB01000046.1_selection_translation_frame_+1

METKSLVTVFLVSCIFLSICLHPVDSQGLRWGREFEEDESPRTRAVKSDYFRRKLNEKKFEKTADKGKQ*

>RDEB01000046.1_selection-1_translation_frame_-1

MTTKTQLALLLLSCAVMAVLIQPVASQKHGLRWGKRESEQWDDYAENLQPYPRYNYENDHNGKPIRLLHNNFS
SHTRI*

>RDEB01000046.1selectionselectionrevtranslationframe+1

MSSSKLCLFFFIFGLLLTIYCQHTEGQLLGIRWGRNYYDPDVTREEYKGNLWESMKRRFSQQDENGGCSKC*

Pocillopora damicornis

>TSA: Pocillopora damicornis contig_4178 transcribed RNA sequence

METKSLVALFLVSCIFVSVCFQPAASQGLRWGREYEEEEEKHRVNPVKADYLRRKEMHRTFEDAADEANFDYN
RR*

>RCHS01000794.1selectionselectiontranslationframe+1

MVSSKAFFVSFLVSCVLLSVYCQKAEEQGLPGIRWGRHFQEDDLTQRGGEGKLWRVMEQKRNHHYRFNGKTCK
DVKKKQHQQQF*

>RCHS01000794.1selectionselection-1translationframe+1

Stylophora pistillata

>GARY01002466.1selectionselectionrevtranslationframe+1

MFLVSCIFLSVCFQPAASQGLRWGREYEEKEEGHRMNPVKADYLKRKEMHRTFEDAAENG*

Porites rus

>Porites rus isolate 14846/IV/SATS-LN/2007 genome assembly, contig:
sscaffold02212, whole genome shotgun sequence

MDAKSLVAVLFVSCLFFSVCLQPAASQGLRWGREFEEEHHRMNPAKADYVRERLYRKRFENSAKKGEKIKAYD
*

Orbicella faveolata

>Orbicella faveolata isolate FL scaffold521_size105876, whole genome shotgun sequence

MEAKSLVVILLVSCVFVSVCLQPASSQGLRWGREFKEENPKIERVKADYLRKKQMRESFDDATEKGEKKIKNK
YFFLKRDL*

Corallimorpharia (see Table 7, neuropeptide family 10)

Amplexidiscus fenestrafer

>scaffold_111selectionselectiontranslationframe-1

MSSSRMLIVFLVVSCVILAMFNGNVEGQRQHGLRWGRSFHVTGAKSEAKLWSLLKRWFSSHQRVGK
FERERKCRFRSQFYRNYNLRLI

>scaffold_111selectionselectiontranslationframe+1

MEAKSLFLVFMVSCVLLSVGLQPVSSQGIRWGREFEEDNPRMDPVKMAYQRRQNRQRSFDEPADVE
RGKTKQHKGEGKLRELVTQLHAAHFHHRL*

Corynactis australis

>gb|GELM01047605.1|selectiontranslationframe+1

MGFKNLALLLFVSCALLSVCLEPVSSQGIRWGREFKQDEPEMSQAKMDFLRRKNSQRSFDDSEEKDFDFHKM*

>gb|GELM01025708.1|selectiontranslationframe-2

MVSKTQLAVLLASCALLSLLVQPAVGQQKGVLRWGKRNTGLSEEPEREDLPSWRQYQERDYNRKDRYRD

Discosoma sp.

>scaffold_89selectionselectiontranslationframe-1

MEAKNLFLVFVVSCVLLSVYLQPVSSQGIRWGREFEEDHPRMDAMKKAYQRMKNRQRSFDESADLERGKAKQH
IGEGRVARICQAPAAHFITGIVILFHSPPLLRPS*

>scaffold_89selectionselectiontranslationframe+1

MSSSRKLIAFLVVSCVILAMFNEKVEGQLPGIRWGRSFQPDDVTGAKSEGKLWSLLKKRFSNHRRVGKSENGR
*

Ricordea yuma

>gb|GELN01026052.1|selectiontranslationframe+2

MESKQIAVFCLVCCVLLYSCFQPASSQGLRWGREFEENHPRMENVPAAKMAFLKRKNNQRRTFEDAAEREIDF
NRM*

>gb|GELN01030591.1|selectiontranslationframe+1

MSSSRRLLLFLVISCFLFSMFNEYVEGQLPGIRWGRNFEYDDVIKGENEGKLWSLFRRWMADHHKTDEKGNKD
LS*

Zoantharia (see Table 7, neuropeptide family 10)

Protopalythoa variabilis

>GCVI01037279.1selectiontranslationframe-2

MTSFGKSLLVFMLVSAFVVLHAPGSTGQGLRWGREFQLDNEGAEKLYYDMKLRELKRRIAAAKEKGYTNA*

>GCVI01064647.1selectiontranslationframe-1

MERTILLACLLMSMAMISFFPPTVSGQMKGIRWGKKSLESSDFSLNEQNLDDDDRFSKEEMAQMKRAFKNYRM
AVKARLLRRQLY*

>GCVI01049254.1selectiontranslationframe+1

MESKRVFAFLLISCVVLMACLHTTEAQGLRWGREYEENGRFRKVFRNNQKRTYDDSNDKDFEKEFGKA*

Zoanthus sp.

>GGTW01123285.1selectiontranslationframe-3

MESKRVLAFLLISCVVLAVLLHTTEAQLGGMRWGREFEENGKLARLLRRYLQRRNFDDSYDKDFEKAFEKA*

>GGTW01112878.1selectiontranslationframe-2

MISRTTKATLLLLGMFALVVLLAPDATGQGLRWGREYELDEDAALFSPLRMKINELRKRLKDGAKKDYSAK*

Ceriantharia (see Table 7, neuropeptide family 10)

Pachycerianthus borealis

>TSA: Pachycerianthus borealis, contig TRINITY_DN13510_c1_g2_i1, transcribed RNA sequence

MACQKTFVMMLVCGLVLSLFSQSEAQAIKGLRWGKRNFEAADEIRRPVAYENSKRGENRIPAVDENENLEWRY
*
