# Supplementary material for: Review: The evolution of peptidergic signaling in Cnidaria and Placozoa, including a comparison with Bilateria
Source: Front Endocrinol (Lausanne). 2022 Sep 23;13:973862. doi: 10.3389/fendo.2022.973862 (PMC9545775; doi:10.3389/fendo.2022.973862)
Supplement: Supplementary file 5 [file Image_5.pdf]

Supplementary Figure 5

FASTA files (A) of three candidate glycoprotein hormone subunits that might be ligands for the *N. vectensis* LGRs belonging to type-A or type-B (see Fig. 6). Signal peptides are underlined and the eleven conserved cysteine residues that are known to form cystine bridges in other glycoprotein hormone subunits are highlighted in yellow. Protein sequences are derived from TrEMBL entries with the following accession numbers: A7RH96 (= Nvec-GPH1) ; A7SFH8 (= Nvec-GPH2); A7RWM9 (= Nvec-GPH3). In (B), these three sequences are aligned with human glycoprotein hormone subunits.

A

```
>Nvec-GPH1
MKLGTLVVLIILSVVLFQPFLNAQASLRFSSAKCRYRNFKQKVQIRDNPLCIPKKIRRSKCVGSCKSSAFPIVSA
KGNQFRKICNCCTPIVEENEIVQFDGCEDTLTLPVIKRCECRPCGTRDEL

>Nvec-GPH2
MGGVSPIYGTLVVALLVIISEMSAEANYKEVCQPRQGSVDVRLTGCPEGKAFLHLCVGTCYTEDNVVRDEASCTC
CKPTKFRSVQVDVECRHNKAWGIVKHVMREHEHCACVPCLG

>Nvec-GPH3
MSVNHVLLLTFLLIVATTTSRGNPFKIAYANQQCKLSGYTMEVTVHSCQPRKISVNTCVGTCVSSALPAAGLRIE
PACTCCQEIESHEVEVGLWCQASPNSAWTQEYHVIKTATKCACRPC
```

B

```
Nvec-GPH1     -MKLGTLVVLIILSVVLFQPFLNAQASLRFSSAKCRYRNFKQKVQIRDNPLCIP-KKIRRSKCVGSCKS
Nvec-GPH2     ---MGGVSPIYGTLVVALLVIISEMSAEANYKEVCQPRQGSVDVRL---TGCPE-GKAFLHLCVGTCYT
Nvec-GPH3     ---VNHVLLLTFLLIVATTTSRGNPFKIAYANQQCKLSGYTMEVTV---HSCQP-RKISVNTCVGTCVS
Hsap-FSH-B    ---------MKTLQFFFLFCCWKAICCNS-----CELTNITIAIEK---EECRFCISINTTWCAGYCYT
Hsap-TSH-B    ------MTALFLMSMLFGLTCGQAMSF-------CIPTEYTMHIER---RECAYCLTINTTICAGYCMT
Hsap-LH-B     ------MEMLQGLLLLLLLSMGGAWASREPLRPWCHPINAILAVEK---EGCPVCITVNTTICAGYCPT
Hsap-CG-B     ------MEMFQGLLLLLLLSMGGTWASREMLRPRCRPINATLAVEK---EGCPVCITVNTTICAGYCPT
Hsap-GPA2     ----MPMASPQTLVLYLLVLAVTEAWGQEAVIPGCHLHPFNVTVRSDRQGTCQ--GSHVAQACVGHCES
Hsap-GPB5    MKLAFLFLGPMALLLLAGYGCVLGASSGNLRTFVGCAVREFTFLAKK---PGCRG-LRITTDACWGRCET
                                                *                *          * * * :

Nvec-GPH1     SAFPI------VSAKGNQFRKICNCCTPIVEENEIVQFDGCED---------TLTLPVIKRCECRPCGT
Nvec-GPH2     EDN------------VVRDEASCTCCKPTKFR-SVQVDVECRHNKAWG--IVKHVMREHEHCACVPCLG
Nvec-GPH3     SALP---------AAGLRIEPACTCCQEIESH-EVEVGLWCQASPNSAWTQEYHVIKTATKCACRPC--
Hsap-FSH-B    RDL--------VYKDPARPKIQKTCTFKELVY-ETVRVPGCAHHADS-----LYTYPVATQCHCGKCDS
Hsap-TSH-B    RDING------KLFLPKYALSQDVCTYRDFIY-RTVEIPGCPLHVAP-----YFSYPVALSCKCGKCNT
Hsap-LH-B     MMR--------VLQAVLPPLPQVVCTYRDVRF-ESIRLPGCPRGVDP-----VVSFPVALSCRCGPCRR
Hsap-CG-B     MTR--------VLQGVLPALPQVVCNYRDVRF-ESIRLPGCPRGVNP-----VVSYAVALSCQCALCRR
Hsap-GPA2     SAFPSRYSVLVASGYRHNITSVSQCCTISGKQ-VTVKLPNCAPGVDP-----FYTYPVAIRCDCGACST
Hsap-GPB5     WEKP-------ILEPPYIEAHHRVCTYNETLK-KVKVQLQCVGSRRE-----ELEIFTARACQCDMCRL
                                      *               *                    * *  *

Nvec-GPH1     RDEL----------------------------------------------
Nvec-GPH2     --------------------------------------------------
Nvec-GPH3     --------------------------------------------------
Hsap-FSH-B    DSTDCTVRGLGPSYCSFGEMKE----------------------------
Hsap-TSH-B    DYSDCIHEAIKTNYCTKPQKSYLVGFSV----------------------
Hsap-LH-B     STSDCGGPKDHPLTCDHPQLSGLLFL------------------------
Hsap-CG-B     STTDCGGPKDHPLTCDDPRFQASSSSKAPPPSLPSPSRLPGPSDTPILPQ
Hsap-GPB5     ATTECETI------------------------------------------
Hsap-GPA2     SRY-----------------------------------------------
```
