# Supplementary material for: Review: The evolution of peptidergic signaling in Cnidaria and Placozoa, including a comparison with Bilateria
Source: Front Endocrinol (Lausanne). 2022 Sep 23;13:973862. doi: 10.3389/fendo.2022.973862 (PMC9545775; doi:10.3389/fendo.2022.973862)
Supplement: Supplementary file 6 [file Image_6.pdf]

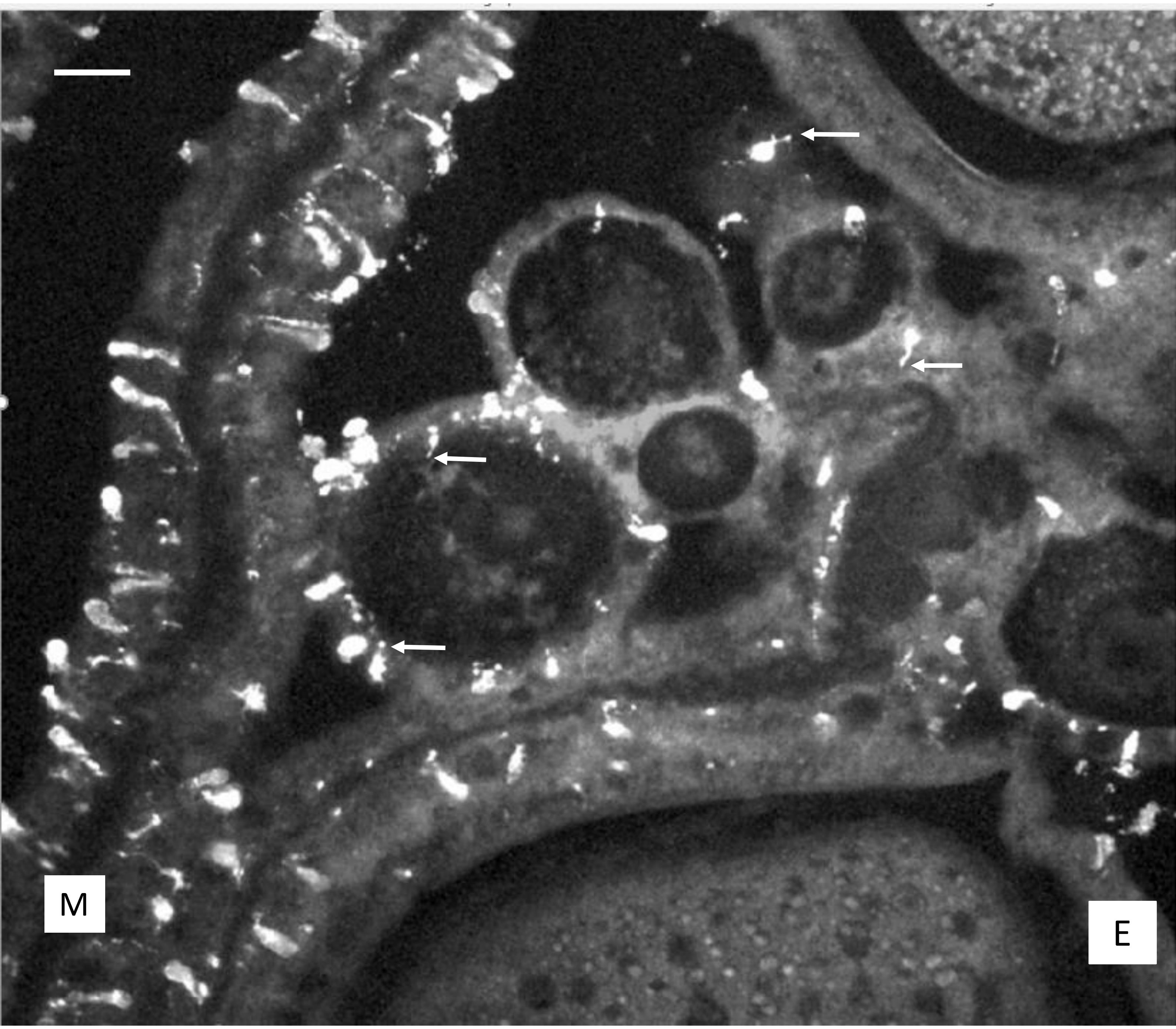

Supplementary Figure S8. This figure is a screenshot from Fig. 9B of Pernet et al. 2004 [60]. It shows the presence of presumed endocrine cells in the endoderm of an ovary from the anthozoan Renilla köllikeri stained with an antiserum against the neuropeptide Antho-RFamide (pGlu-Gly-Arg-Phe-amide). Please note the apical cell protrusions on the immunostained endocrine-like cells (arrows) and the absence of a clear nervous system. M= mesoglea; E= endoderm; bar = 50 micrometer.
