# Supplementary material for: Review: The evolution of peptidergic signaling in Cnidaria and Placozoa, including a comparison with Bilateria
Source: Front Endocrinol (Lausanne). 2022 Sep 23;13:973862. doi: 10.3389/fendo.2022.973862 (PMC9545775; doi:10.3389/fendo.2022.973862)
Supplement: Supplementary file 7 [file Table_1.pdf]

Supplementary Table 1

Accession numbers of the GPCR sequences used to calculate the phylogenetic tree of Figure 2.

Label in Fig. 2	Swiss-prot	FlyBase	our Tc numbers
Dm-AKHR	Q7KTL9	CG11325	
Dm-mGluR	P91685	CG11144	
Dm-NPFR	Q9VNM1	CG1147	
Dm-NTLR	P30974	CG6515	
Dm-SKR2	Q7M3J6	CG6881	
Dm-SKR1	A8JUP8	CG6857	
Dm-TKR	P30975	CG7887	
Tc-AKHR1	Q1W7L1		Tc46
Tc-AKHR2	N1NVE7		Tc47
Tc-ITR	B1NWV5		Tc45
Tc-NPFR	A0A139WBY5		Tc59
Tc-NTLR	D6WD17		Tc41
Tc-SKR1	D2A204		Tc34
Tc-SKR2	M9Z5C3		Tc35
Tc-TKR	D6WV75		Tc40
Hs-AVPR1A	P37288		
Hs-AVPR1B	P47901		
Hs-AVPR2	P30518		
Hs-CCKAR	P32238		
Hs-CCKBR	P32239		
Hs-GnRHR	P30968		
Hs-NK1R	P25103		
Hs-NK2R	P21452		
Hs-NK3R	P29371		
Hs-NPY1R	P25929		
Hs-NPY2R	P49146		
Hs-NPY4R	P50391		
Hs-NPY5R	Q15761		
Hs-OTR	P30559		
