# Supplementary material for: Review: The evolution of peptidergic signaling in Cnidaria and Placozoa, including a comparison with Bilateria
Source: Front Endocrinol (Lausanne). 2022 Sep 23;13:973862. doi: 10.3389/fendo.2022.973862 (PMC9545775; doi:10.3389/fendo.2022.973862)
Supplement: Supplementary file 8 [file Table_2.pdf]

Supplementary Table 2

Accession numbers of the sequences used in Figure 6.

Name in Fig. 6	Swiss-prot	GenBank	TrEMBL	CoGe
Dmel-mGluR	P91685			
Hsap-LGR1	P23945			
Hsap-LGR2	P22888	EAX00192		
Hsap-LGR3	P16473			
Hsap-LGR4	Q9BXB1	BAD92980		
Hsap-LGR5	O75473	AAH96324		
Hsap-LGR6	Q9HBX8	BAB39854		
Hsap-LGR7	Q9HBX9	AAG17167		
Hsap-LGR8	Q8WXD0	NP_570718		
Nvec-LGR1			A7RGG2	
Nvec-LGR2		XP_032218699		
Nvec-LGR3			A7RZD5	
Nvec-LGR4			A7SU96	
Nvec-LGR5			A7RJD4	
Nvec-LGR6			A7RJD5	
Nvec-LGR7			A7T202	
Nvec-LGR8			A7SJN7	
Nvec-LGR9			A7RW36	
Nvec-LGR10			A7RQR1	
Nvec-LGR11			A7RMT7	
Nvec-LGR12			A7RYQ5	
Nvec-LGR13			A7RYQ4	
Tadh-LGR1		XP_002107856		TriadITZ_000533
Tadh-LGR2		XP_002107746		TriadITZ_000292
Tadh-LGR3		XP_002107856		TriadITZ_000519
Tadh-LGR4		XP_002112613		TriadITZ_005432
Tadh-LGR5		XP_002114819		TriadITZ_007850
Tadh-LGR6				TriadITZ_009725
Tadh-LGR7				TriadITZ_009708
Tadh-LGR8				TriadITZ_010350
Tadh-LGR9				TriadITZ_009512
Tadh-LGR10				TriadITZ_009711
Tadh-LGR11				TriadITZ_008579
Tadh-LGR12				TriadITZ_010180
Tadh-LGR13				TriadITZ_010178
Tadh-LGR14				TriadITZ_000534
Tadh-LGR15				TriadITZ_000533
Tadh-LGR16				TriadITZ_000554
Tadh-LGR17				TriadITZ_001554
Tadh-LGR18				TriadITZ_002654
Tadh-LGR19				TriadITZ_010559

Tadh-LGR20				TriadITZ_010558
Tadh-LGR21				TriadITZ_005220
Tadh-LGR22				TriadITZ_005222
Tadh-LGR23				TriadITZ_005226
Tadh-LGR24				TriadITZ_005271
Tadh-LGR25				TriadITZ_005258
Tadh-LGR26				TriadITZ_002476
Tadh-LGR27				TriadITZ_002474
Tadh-LGR28				TriadITZ_002473
Tadh-LGR29				TriadITZ_010632
Tadh-LGR30				TriadITZ_010631
Tadh-LGR31				TriadITZ_010630
Tadh-LGR32				TriadITZ_010628
Tadh-LGR33				TriadITZ_010867
Tadh-LGR34				TriadITZ_010608
Tadh-LGR35				TriadITZ_010610
Tadh-LGR36				TriadITZ_010609
Tadh-LGR37				TriadITZ_009009
Tadh-LGR38				TriadITZ_009008
Tadh-LGR39				TriadITZ_009007
Tadh-LGR40				TriadITZ_010145
Tadh-LGR41				TriadITZ_005060
Tadh-LGR42				TriadITZ_009084
Tadh-LGR43				TriadITZ_009081
Tadh-LGR44				TriadITZ_009123
Tadh-LGR45				TriadITZ_002813
Tadh-LGR46				TriadITZ_002580
