# Supplementary material for: Review: The evolution of peptidergic signaling in Cnidaria and Placozoa, including a comparison with Bilateria
Source: Front Endocrinol (Lausanne). 2022 Sep 23;13:973862. doi: 10.3389/fendo.2022.973862 (PMC9545775; doi:10.3389/fendo.2022.973862)

Supplementary Figure 2

Circular phylogenetic tree of all family-A (rhodopsin-like) GPCRs from *H. sapiens* (green), *N. vectensis* (blue), and *T. adherens* (red). Human olfactory receptors were excluded from the analysis. Bootstrap values are given next to the braches. The names of the human GPCRs or receptor clades are also added. Biogenic amine receptors include the receptors for dopamine, adrenaline, serotonin, histamine, the muscarinic acetylcholine receptors, and the trace amine receptors. Abbreviations sorted according to their clockwise positions: GRP, gastrin releasing peptide; ET, endothelin; NMU, neuromedin U; NT, neurotensin; TRH, thyrotropin releasing hormone; NPY, neuropeptide Y; NPFF, Neuropeptide FF; PrRP, prolactin releasing peptide; CCK, cholecystokinin; NK, neurokinin; PARs, protease activated receptors; HCA-Rs, hydroxycarboxylic acid receptors; SST, somatostatin; NP-BW, neuropeptide BW; MCH, melanin concentrating hormone; VP, vasopressin; OT, oxytocin; UT, urotensin; AT, angiotensin; BK, bradykinin; GnRH, gonadotropin releasing hormone; NPS, neuropeptide S; FSH, follicle stimulating hormone; TSH, thyroid stimulating hormone; LH/CG, luteinizing hormone/choriogonadotropin; LGRs, leucine rich repeats containing G protein-coupled receptors.

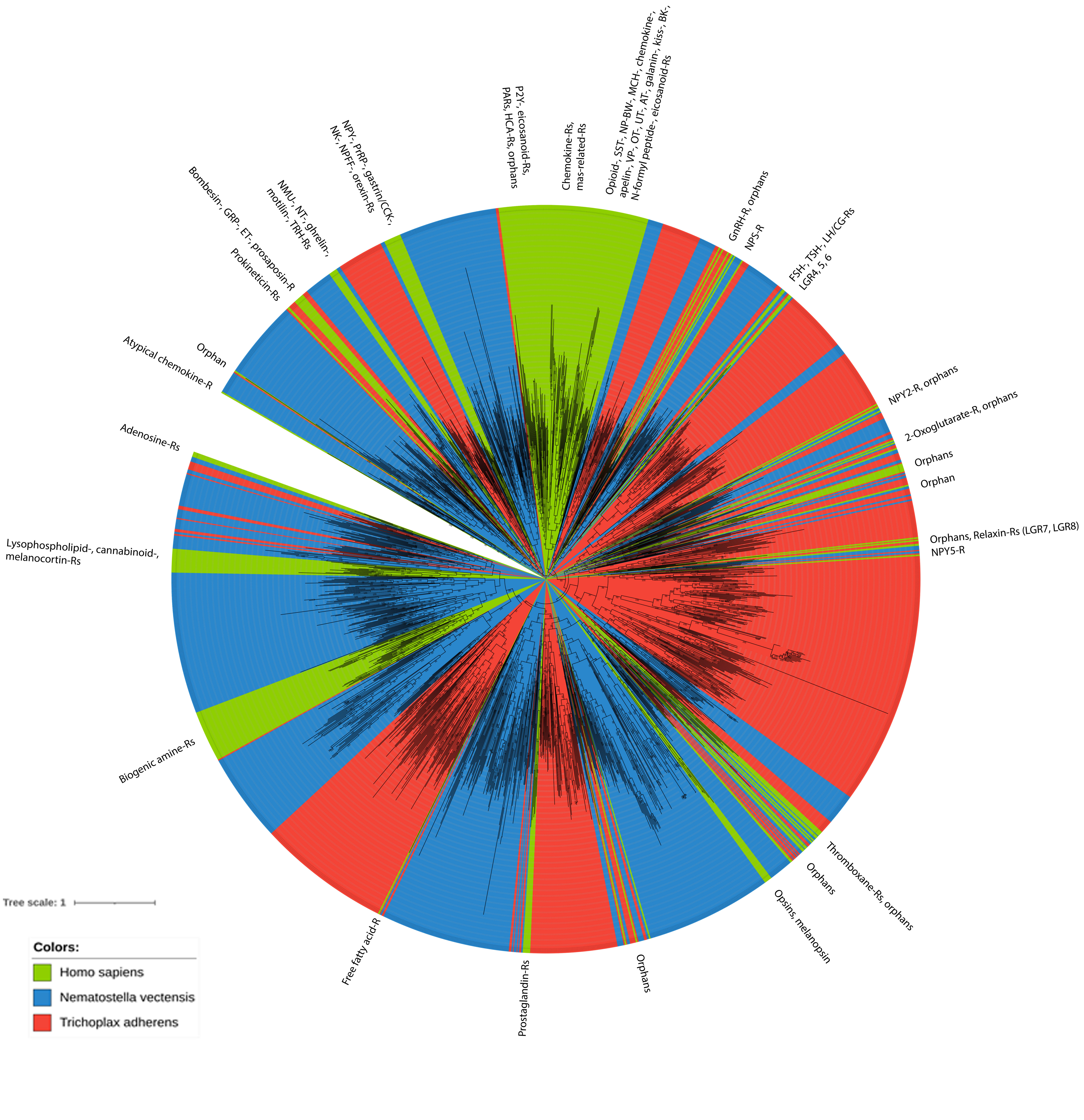

Supplement: Supplementary file 2 [file Image_2.pdf]
